# Transcriptional responses to direct and indirect TGFB1 stimulation in cancerous and noncancerous mammary epithelial cells

**DOI:** 10.1186/s12964-024-01821-5

**Published:** 2024-10-28

**Authors:** Patryk Janus, Paweł Kuś, Roman Jaksik, Natalia Vydra, Agnieszka Toma-Jonik, Michalina Gramatyka, Monika Kurpas, Marek Kimmel, Wiesława Widłak

**Affiliations:** 1 Maria Skłodowska-Curie National Research Institute of Oncology, Gliwice Branch, Wybrzeże Armii Krajowej 15, Gliwice, 44-102 Poland; 2 Department of Systems Biology and Engineering, Silesian University of Technology, Akademicka 16, Gliwice, 44-100 Poland; 3 Departments of Statistics and Bioengineering, Rice University, Houston, TX USA

**Keywords:** Bystander effect, Cell death, EMT, Cell cycle, Estrogen signaling, Mammary epithelial cells, MCF10A, MCF7, TGFβ signaling

## Abstract

**Background:**

Transforming growth factor beta (TGFβ) is important for the morphogenesis and secretory function of the mammary gland. It is one of the main activators of the epithelial–mesenchymal transition (EMT), a process important for tissue remodeling and regeneration. It also provides cells with the plasticity to form metastases during tumor progression. Noncancerous and cancer cells respond differently to TGFβ. However, knowledge of the cellular signaling cascades triggered by TGFβ in various cell types is still limited.

**Methods:**

MCF10A (noncancerous, originating from fibrotic breast tissue) and MCF7 (cancer, estrogen receptor-positive) breast epithelial cells were treated with TGFB1 directly or through conditioned media from stimulated cells. Transcriptional changes (via RNA-seq) were assessed in untreated cells and after 1–6 days of treatment. Differentially expressed genes were detected with DESeq2 and the hallmark collection was selected for gene set enrichment analysis.

**Results:**

TGFB1 induces EMT in both the MCF10A and MCF7 cell lines but via slightly different mechanisms (signaling through SMAD3 is more active in MCF7 cells). Many EMT-related genes are expressed in MCF10A cells at baseline. Both cell lines respond to TGFB1 by decreasing the expression of genes involved in cell proliferation: through the repression of *MYC* (and the protein targets) in MCF10A cells and the activation of p63-dependent signaling in MCF7 cells (*CDKN1A* and *CDKN2B*, which are responsible for the inhibition of cyclin-dependent kinases, are upregulated). In addition, estrogen receptor signaling is inhibited and caspase-dependent cell death is induced only in MCF7 cells. Direct incubation with TGFB1 and treatment of cells with conditioned media similarly affected transcriptional profiles. However, TGFB1-induced protein secretion is more pronounced in MCF10A cells; therefore, the signaling is propagated through conditioned media (bystander effect) more effectively in MCF10A cells than in MCF7 cells.

**Conclusions:**

Estrogen receptor-positive breast cancer patients may benefit from high levels of TGFB1 expression due to the repression of estrogen receptor signaling, inhibition of proliferation, and induction of apoptosis in cancer cells. However, some TGFB1-stimulated cells may undergo EMT, which increases the risk of metastasis.

**Supplementary Information:**

The online version contains supplementary material available at 10.1186/s12964-024-01821-5.

## Abstract (plain language summary)

Transforming growth factor beta (TGFβ) is a multifaceted cytokine that controls numerous physiological and pathological processes during development and carcinogenesis. Its best-known function is the activation of epithelial–mesenchymal transition (EMT), a process crucial for tissue remodeling and regeneration. During EMT, epithelial cells lose their connections to adjacent cells and acquire mesenchymal characteristics, such as migratory ability. Compared with cancer cells, normal (nontumorigenic) cells usually respond differently to TGFβ stimulation. Typically, TGFβ inhibits the proliferation of epithelial cells and may promote cell death. In cancer cells, TGFβ often promotes tumor progression. TheTGFβ-mediated induction of EMT increases cell mobility, which is associated with the formation of metastases and tumor invasion.

In this work, we compared the transcriptional response of noncancerous (MCF10A) and cancerous (MCF7; estrogen receptor-positive) breast epithelial cells to direct stimulation by TGFB1 and its indirect effect through conditioned media. Some of TGFB1-induced changes, including inhibition of proliferation and induction of EMT, were similar in both cell lines. However, these changes were associated with different upstream signaling pathways. Other changes were more specific, such as disruption of estrogen-related signaling or induction of cell death in MCF7 cells. Direct incubation with TGFB1 and treatment of cells with conditioned media similarly affected target cells, indicating the presence of a bystander effect. Moreover, transcript profiling by RNA-seq revealed that the TGFβ signaling pathway is already active in untreated MCF10A cells, which may be due to their origination from a fibrotic lesion.

**Supplementary Information:**

The online version contains supplementary material available at 10.1186/s12964-024-01821-5.

## Background

The mammary gland undergoes successive changes during pregnancy, lactation, and involution. These changes are largely governed by the coordinated action of reproductive hormones and growth factors. Transforming growth factor beta (TGFβ) is critically important for mammary morphogenesis and secretory function through specific regulation of epithelial cell proliferation, apoptosis, and the extracellular matrix [1]. It is also secreted into colostrum and milk (along with other cytokines) necessary for the maturation of the immune system in the developing gastrointestinal tract of the newborn [2]. TGFβ exists in three different isoforms (TGFβ1, TGFβ2, and TGFβ3; official symbols TGFB1, TGFB2, and TGFB3, respectively) and is secreted by many cell types in a latent form and activated extracellularly. By acting on its cell membrane receptor(s) (TGFBR1, TGFBR2, and TGFBR3; [3]), TGFβ activates different signaling cascades [4]. Canonical TGFβ signaling involves the activation of the SMAD (R-SMADs, Co-SMAD, I-SMADs) pathway [5]. Activated by phosphorylation, R-SMADs (receptor-regulated SMADs, i.e., SMAD2 and SMAD3) form complexes with Co-SMAD (common partner SMAD, i.e., SMAD4). They accumulate in the nucleus and can induce cell type-specific gene expression profiles by interacting with specific subsets of other transcription factors, coactivators, and corepressors. I-SMADs (inhibitory SMADs, i.e., SMAD6 and SMAD7) are part of the feedback loops. They are induced by TGFβ signaling and act by competing with R-SMADs for receptor binding, thereby inhibiting R-SMAD phosphorylation. In contrast, SMAD-independent pathways (e.g., through activation of MAPK family members, TRAF4/6, NFκB, PI3K-AKT, RHO-like GTPases, etc.) can initiate parallel signaling that ultimately cooperates with SMAD or crosses over to other major signaling pathways (such as estrogen, EGF, HGF, WNT ligands, etc.) [6]. The net activation of SMAD-dependent and SMAD-independent pathways, and the interactions derived from the presence or absence of other parallel signaling cascades determine the functional response to TGFβ [7].

TGFβ is one of the main activators of epithelial–mesenchymal transition (EMT) [8]. EMT is a process by which epithelial cells lose their basoapical polarity and cell–cell adhesion and acquire mesenchymal characteristics, such as an enhanced ability to migrate and invade surrounding tissues. This process is coordinated by multiple transcription factors, including SNAI1, SNAI2, ZEB1, and ZEB2, and is characterized by the loss of epithelial cell markers such as E-cadherin (cadherin 1, CDH1). This is followed by an increase in the expression of mesenchymal cell markers such as N-cadherin (cadherin 2, CDH2), vimentin (VIM) and fibronectin (FN1) [9, 10]. These changes lead to a reduction in adhesion between cells and an increase in the secretion of enzymes (mainly metalloproteinases, MMPs) that degrade the extracellular matrix. EMT occurs mainly during embryogenesis (type 1 EMT) but is also associated with the regeneration of adult tissues, wound healing, and fibrosis (type 2 EMT). In addition, during tumor progression, activation of EMT (type 3 EMT) provides cells with the plasticity to form metastases [11]. These altered cells can detach from the original tumor mass, infiltrate surrounding tissue, and enter blood vessels. After leaving vessels at a distant site, cancer cells, depending on the local microenvironment, either start to proliferate and revert to a more epithelial phenotype or remain in a dormant stage for a longer time, thus allowing the disease to recur [12]. In both cases, it leads to metastatic disease, the leading cause of death in cancer patients. The properties of the stem cells that develop during EMT can also make them resistant to therapies. Epithelial–mesenchymal interactions are important for normal mammary gland development and for breast tumorigenesis [13, 14].

In normal cells, TGFβ inhibits proliferation, modulates the extracellular matrix, induces differentiation or fibrosis, or promotes apoptosis. When cancer is initiated, TGFβ can inhibit tumor growth and thus may be a tumor suppressor [15]. However, tumor cells often lose their ability to respond to antiproliferative TGFβ signals. Thus, in later stages of cancer, TGFβ signaling is thought to promote tumor progression. Importantly, the secretion of TGFβ is increased in many types of tumors, which enhances mobility and induces EMT of cancer cells. It also affects surrounding stromal, immune, endothelial, and smooth muscle cells. As a result, it enables extracellular matrix remodeling and metastasis, and causes immunosuppression and angiogenesis, increasing the invasiveness of cancer [5, 16].

TGFβ signaling has been extensively studied in many developmental contexts, including the induction of EMT. While the cellular and molecular hallmarks of EMT progression (such as loss of cell adhesion and nuclear localization of catenin beta 1) are straightforward, the cellular signaling cascades that result in EMT remain less clear. To investigate TGFβ signaling and downstream transcriptional responses in noncancerous and cancerous cells derived from the breast epithelium, MCF10A and MCF7 cells were stimulated directly with TGFB1 or exposed to conditioned media from TGFB1-stimulated cells. We found that TGFβ signaling through TGFBR2 (TGFβ receptor type II), SMAD2 (receptor-regulated SMAD), SNAI2, and ZEB2 may be more important for maintaining the fibrocystic phenotype of the MCF10A cell line than the cancerous phenotype of MCF7 cells. On the other hand, signaling through SMAD3 is more active in MCF7 cells. TGFB1 induced EMT in both cell lines and inhibited proliferation via different mechanisms: through the repression of MYC (and its targets) in MCF10A cells and through p63-dependent signaling in MCF7 cells. Finally, inhibition of estrogen signaling and cell death were observed in MCF7 cells after treatment. More importantly, conditioned media also had a major effect on the transcriptome, especially in MCF10A cells. The effect was much weaker in MCF7 cells.

## Methods

### Cell lines and treatments

Nontumorigenic human breast epithelial MCF10A cells (authenticated in 2016) were cultured in DMEM/F12 medium supplemented with 5% horse serum (BioWest, Nuaillé, France), 5 µg/ml insulin (Sigma‒Aldrich, Saint Louis, MO, USA), 0.5 µg/ml hydrocortisone (Sigma‒Aldrich), and 20 ng/ml EGF (Sigma‒Aldrich). Breast cancer MCF7 cells (authenticated in 2016) were cultured in DMEM/F12 medium supplemented with 10% fetal bovine serum (EURx, Gdansk, Poland). The cells were routinely tested for mycoplasma contamination. Cells were seeded onto culture plates the day before the start of the experiment (at different densities to achieve ~ 80% confluency at the time of cell harvesting and RNA/protein isolation). Two different recombinant human TGFB1 cytokine (#100–21 C, PeproTech EC Ltd., London, UK; at calculated final concentration 10 ng/ml) treatment protocols were applied depending on further analyses: (i) for continuous treatment, starting from day 1, half of the medium was replaced daily with fresh TGFB1 for 24 h; (ii) for pulsed treatment, each day, TGFB1 was administered in fresh medium for 2 h and then the medium was changed to fresh medium for another 22 h to obtain conditioned medium (CM).The CM from each treatment day was used to treat other cells to induce potential bystander effects (Figure S2a).

### Protein extraction and western blotting

Whole-cell extracts were prepared using RIPA buffer supplemented with Complete™ protease inhibitor cocktail (Roche, Indianapolis, IN, USA) and PhosStop™ phosphatase inhibitor (Roche). To isolate the nuclear extracts, the cells were first lysed for 10 min at 4 °C in 20 mM Tris (pH 7.6), 10 mM KCl, 2 mM MgCl_2_, 1 mM DTT, 0.5 mM EGTA, 0.5% NP40, and 2.5% glycerol to release the cell nuclei. After centrifugation for 10 min at 4 °C and 1,700 rpm, nuclear extracts were prepared using RIPA buffer as described above. Proteins (15–30 µg) were separated on SDS‒PAGE gels and blotted onto 0.45 μm–0.22 μm pore nitrocellulose filters (GE Healthcare, Europe GmbH, Freiburg, Germany) using the Trans-Blot Turbo system (Thermo Scientific™ Pierce™ G2 Fast Blotter) for 10 min. Primary antibodies against phospho-SMAD3 (1:1,000, #9520, Cell Signaling Technology, CST, Danvers, MA, USA), SNAI1 (1:2,000, #3879, CST), SNAI2 (1:2,000, #9585, CST), CDH1 (1:4,000, #3195, CST), PARP1 (1:1,000, #9542, CST), cleaved PARP1 (1:1,000, #5625, CST), cleaved Caspase-7 (1:1,000, #8438, CST), cleaved Caspase-9 (1:1,000, #52873, CST), VIM (1:1,000, #5741, CST), ESR1/ERα (1:2,000, #8644, CST), HSPA8/HSC70 (1:5,000, #sc-7298, Santa Cruz Biotechnology, Dallas, TX, USA), and ACTB (1:10,000, #A3854, Merck KGaA) were used. The primary antibody was detected by an appropriate secondary antibody conjugated with horseradish peroxidase (Thermo Fisher Scientific, Waltham, MA, USA) and visualized by an enhanced chemiluminescence (ECL) kit (Thermo Fisher Scientific) or WesternBright Sirius kit (Advansta, Menlo Park, CA, USA). Imaging was performed on X-ray film or with a digital G: BOX imaging system (Syngene, Cambridge, UK).

### Measurement of TGFB1 levels by ELISA

The media from cells treated as shown in Figure S2a were collected, centrifuged (2 min, 2,000 rpm), aliquoted on an ice bath, and then frozen at − 80 °C. For ELISA, the collected media were thawed in an ice bath. The concentration of TGFB1 was determined according to the manufacturer’s instructions (#88-8350-22, Invitrogen, Waltham, Massachusetts, USA). All incubation steps were performed with shaking (200 rpm on a rotary shaker). Measurements were performed on the SPARK spectrophotometer (#1902008176, Tecan, Männedorf, Switzerland). For analysis of differences between compared groups, the student’s T-test was performed.

### Global gene expression profiling and analysis

Total RNA was isolated using the Direct-ZolTM RNA MiniPrep Kit (Zymo Research, Irvine, CA, USA) and digested with DNase I (Worthington Biochemical Corporation, Lakewood, NJ, USA). For each experimental point, RNA from three biological replicates was first tested by RT‒qPCR to determine the efficiency of the treatments. cDNA libraries were sequenced on an Illumina NovaSeq 6000 (run type: paired-end, read length: 2 × 150 bp). Data quality control was conducted using RSeQC [17], FastQC [18], FastQ Screen [19], SAMtools [20], Picard [21], and a set of our custom data analysis and visualization tools. Adapter sequences and low-quality bases (Phred < 20) were trimmed using TrimGalore (v. 0.4.2) with cutadapt (v. 2.10). Trimmed reads shorter than 20nt were discarded. Processed reads (on average 62 M/sample) were aligned to the GRCh38 reference genome using STAR (v. 2.7.9a) [22], with GENCODE transcript database v. 38 (comprehensive, primary regions). The average alignment rate for all samples was 97.7%. Read counts for individual genes were obtained using featureCounts from the Subread package (v. 2.0.0) [23]. Count data for 84 RNA-seq libraries (data deposited in the Array Express collection; acc. E-MTAB-13865) were loaded into R (v. 4.3.2). The count matrix was filtered using the filterByExpr function from the edgeR package (v. 3.44.4, samples grouped by collection day) [24], and differentially expressed genes were detected with DESeq2 (v. 1.40.2) [25]. Finally, the p values were corrected for multiple testing using the Benjamini and Hochberg method. Volcano plots were generated using the ggplot2 package (v. 3.4.3) [26]. For gene set enrichment analysis, we selected a collection of “hallmark” gene sets from the Molecular Signature Database (MSigDB) (v. 2023.1) [27]. Genes were ordered according to their p value and tested for enrichment using the CERNO test [28] from the tmod package (v. 0.50.13) [29]. The most significant results (effect size > 0.7, p value < 0.001 for at least one comparison) of the gene set enrichment analysis were presented using the ggplot2 and/or *tmodPanelPlot* functions from tmod. Upstream regulators were predicted using ChIP-X Enrichment Analysis Version 3 (ChEA3) [30] based on the ReMap transcriptional regulators library constructed from human data. Data on KEGG graphs were rendered by R pathview (v. 1.40.0) [31].

### Expression time trend analysis

Plots of gene expression over time were plotted using DESeq2::*vst - normalized counts* and the ggplot2 R package. Clustering analysis of the expression time trends was performed for genes whose expression was significantly altered (absolute log-fold change: log2FC > 1 and adjusted p value: padj < 0.05) in at least 4 differential expression tests out of 12 comparing expression levels on days 1–6 after the TGFB1 stimulation of MCF7/MCF10A cells. To generate the heatmap, the DESeq2::*vst normalized counts* were scaled and centered using the base R *scale* function in each cell type separately. Finally, the time trend heatmap was plotted using the ComplexHeatmap R package (v. 2.16.0).

## Results

### Activation of the TGFB1-induced signaling pathway in MCF10A and MCF7 cells

MCF10A nontumorigenic breast epithelial cells (isolated from the mammary gland of a patient with fibrocystic disease) and MCF7 breast adenocarcinoma cells (estrogen receptor-positive, ER+) were selected for the study of TGFB1 (a key member of the TGFβ superfamily) signaling. Both cell lines are epithelial and are characterized by CDH1 expression (Figure S1a). TGFβ acts through specific receptors to activate intracellular pathways leading to the phosphorylation of SMAD2/3 proteins. Therefore, to monitor the activation of the pathways triggered by TGFB1, we assessed the level of SMAD3 phosphorylation at Ser423/425 (Figure S1b-d). TGFB1 concentration of 10 ng/ml was the most effective (Figure S1b), so it was chosen for further experiments (it is worth noting, however, that later determinations of active TGFB1 using the ELISA test showed concentrations an order of magnitude lower; Figure S2b). TGFB1 treatment induced typical morphological changes (such as lossening of cell-cell contacts and shape elongation) in both cell lines, with MCF7 cells additionally undergoing changes leading to cell death (PARP1 cleavage was observed, indicating caspase activation; Figure S1c, d) and senescence. Pilot experiments showed strong phosphorylation and nuclear accumulation of SMAD3 after just one hour of TGFB1 treatment. Only in MCF10A cells was this accompanied by the significant nuclear accumulation of the SNAI1 and SNAI2 transcription factors (which are known to promote *CDH1* repression; [32–34]). In addition, experiments showed that in MCF10A cells, the response to TGFB1 treatment manifested by SMAD3 phosphorylation was extinguished over the next few hours. On the other hand, in MCF7 cells, SMAD3 phosphorylation remained constant for at least 24 h (until a new dose of TGFB1 was administered) (Figure S1c, d). These initial experiments showed that noncancerous and cancerous breast epithelial cells (MCF10A and MCF7, respectively) responded differently to TGFB1 stimulation. Therefore, we performed gene expression profiling to further clarify the differences between the two cell lines. We treated the cells for six consecutive days, with daily administration of TGFB1 for two hours (pulsed treatment). The TGFB1-containing medium was changed to fresh medium, which was collected after another 22 h (so-called conditioned medium, CM) and could be administered to other cells (according to the scheme shown in Figure S2a). This enabled us to assess the ability of extracellularly secreted mediators to induce a response in naïve cells (bystander effect). Interestingly, ELISA test demonstrated that only the medium collected from MCF7 cells showed an approximately threefold increase in TGFB1 levels after such pulsed TGFB1 treatment (Figure S2b) and when administered to subsequent cells was able to activate SMAD3 (Figure S1b). This suggests that the response to conditioned medium in MCF10A cells may be determined by mediators other than TGFB1. Cells responded to both TGF and CM treatment with relaxation of contacts between each other and changes in morphology. Polarized cells forming lamellipodia were typically observed in MCF10A cells from day 1, elongated cell shape from day 3 in both lines, and dead and senescent cells were typical of MCF7 cells (Figure S2c). RNA-seq analyses were performed on cells either directly treated with TGFB1 or treated with conditioned media for 1, 2, 3, 4, 5, or 6 days.

### Comparison of transcriptional responses to direct and indirect stimulation with TGFB1

**Fig. 1:**
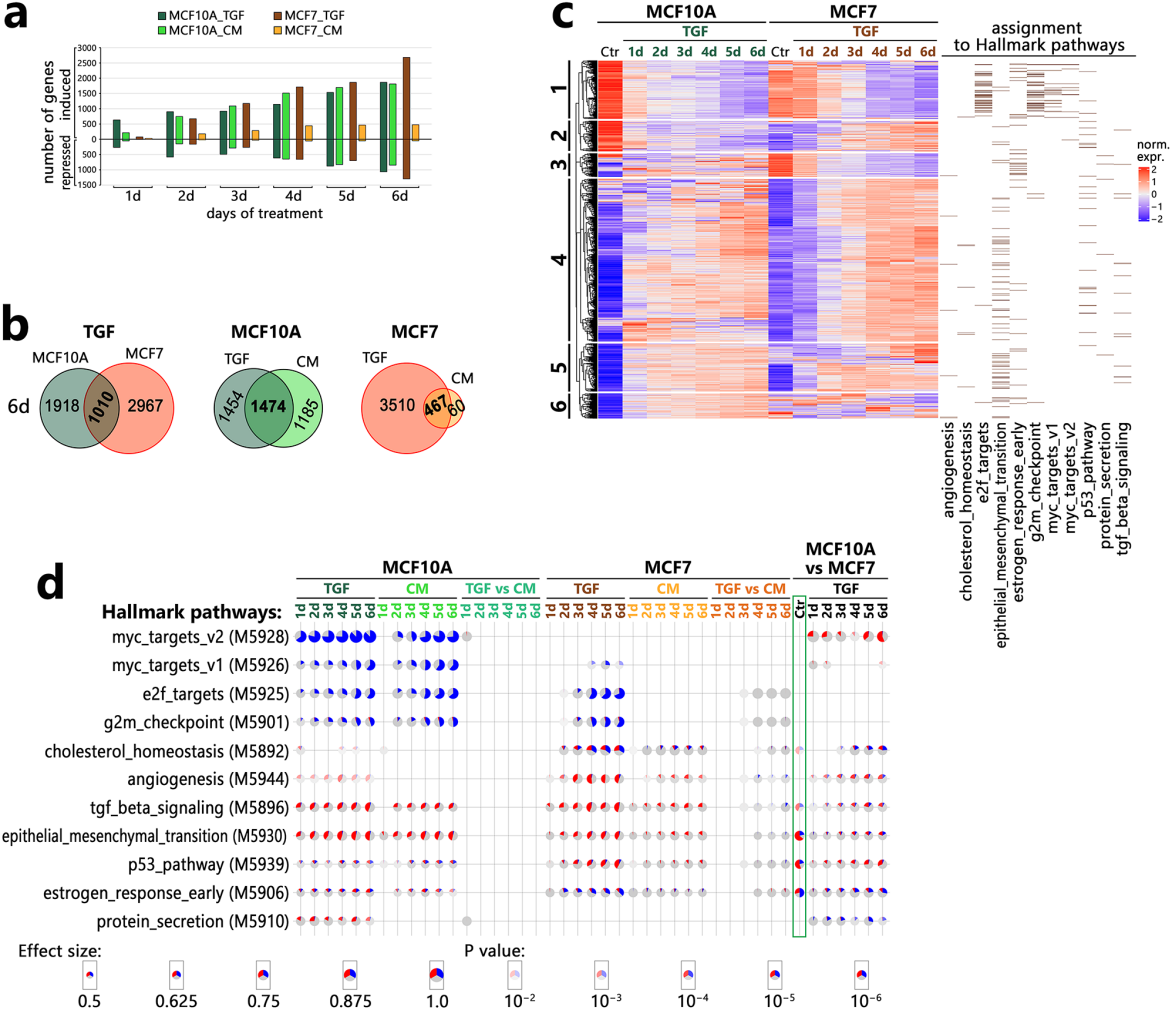
Changes in the global transcriptional profiles of MCF10A and MCF7 cells directly stimulated with recombinant TGFB1 (TGF) or exposed to conditioned media (CM). (**a**) Number of genes induced (log2FC > 1.0, padj < 0.05) or repressed (log2FC < − 1.0, padj < 0.05) after TGF or CM treatment. (**b**) Overlap of genes with altered expression after 6 days of TGF and/or CM treatment in both cell lines (see also Figure S3). (**c**) Heatmap with hierarchical clustering of normalized read counts from RNA-seq (row z-score) for selected genes (1,685) with altered expression after TGF treatment and their assignment to hallmark pathways (on the right). (**d**) Gene set enrichment analysis showing significant pathways from the hallmark gene set collection detected in MCF10A and MCF7 cells treated with TGF or CM, as well as differences between treatments and differences between cell types: untreated (Ctr, shown in green rectangle) and in response to TGF (first compared to corresponding Ctr). The sizes of the pie charts correspond to the effect size, while color intensity corresponds to the p value; blue and red indicate the fractions of downregulated and upregulated genes, respectively. The cells were treated according to the scheme shown in Figure S2a

To compare the effects of direct TGFB1 treatment and exposure to CM, we assessed changes in gene expression relative to that in untreated cells (day 0, Ctr) (Supplementary data 1). Initially, the number of affected genes (change at least 2-fold, padj < 0.05) was significantly greater (several-fold) in MCF10A cells than in MCF7 cells, but after six days of treatment, the effect was stronger in MCF7 cells (Figs. 1a, b and S3a). Only part of this gene pool was common in both cell lines (Figs. 1b and S3a). The response to CM (assessed as the number of affected genes) was delayed compared to the response to direct TGFB1 treatment in both cell lines. However, it reached the same level by day three in MCF10A cells, while it was stabilized at a low level in MCF7 cells (Fig. 1a). The poor transcriptional response to conditioned media in MCF7 cells putatively resulted from cell death induced after TGFB1 treatment, which reduced the number of mediator-secreting cells (as suggested by PARP1 cleavage; see Figures S1c, d, and S5b). Nevertheless, 46.9–55.4% of genes in CM-stimulated MCF10A cells and 80-88.6% in CM-stimulated MCF7 exhibited similar changes in expression as upon direct TGFB1 treatment (Figs. 1b and S3b).

Hierarchical clustering analysis based on expression trends identified six clusters of genes affected by TGFB1 in MCF10A and MCF7 cells (Fig. 1c) (Supplementary data 2). Genes activated in both cell lines were grouped into clusters 4 and 5. These gene sets were putatively regulated by, among others, the SMAD3 and SMAD4 transcription factors (prediction based on ChEA3 transcription factor enrichment analysis [30]) (Figure S4a), which were also identified as major upstream regulators in the analysis of all genes activated after treatment (Figure S4b). A similar pattern was visible in cluster 6 but only in MCF10A cells. Assignment to terms from the hallmark collection showed that these clusters were enriched in genes primarily associated with the epithelial_mesenchymal_transition (M5930) (Fig. 1c). On the other hand, cluster 1 contained genes repressed after treatment in both cell lines (and predicted to be regulated by MYC and E2F family of transcription factors; Figure S4a). This cluster was enriched in genes related to the e2f_targets (M5925) and g2m_checkpoint (M5901), which both reflect cell proliferation ability, as well as myc_targets_v1 (M5926) and myc_targets_v2 (M5928). Clusters 2 and 3 showed the opposite pattern in both cell lines; i.e., repression of expression in one cell line was accompanied by activation in the other. Cluster 3 mainly showed inhibition of the expression of genes related to the estrogen_response_early (M5906) in the MCF7 line (Fig. 1c). Moreover, estrogen receptor alpha (ESR1/ERα) was found to be an upstream regulator of this set of genes (Figure S4a), as well as the entire set of genes downregulated after TGFB1 treatment in MCF7 cells (Figure S4b).

**Fig. 2:**
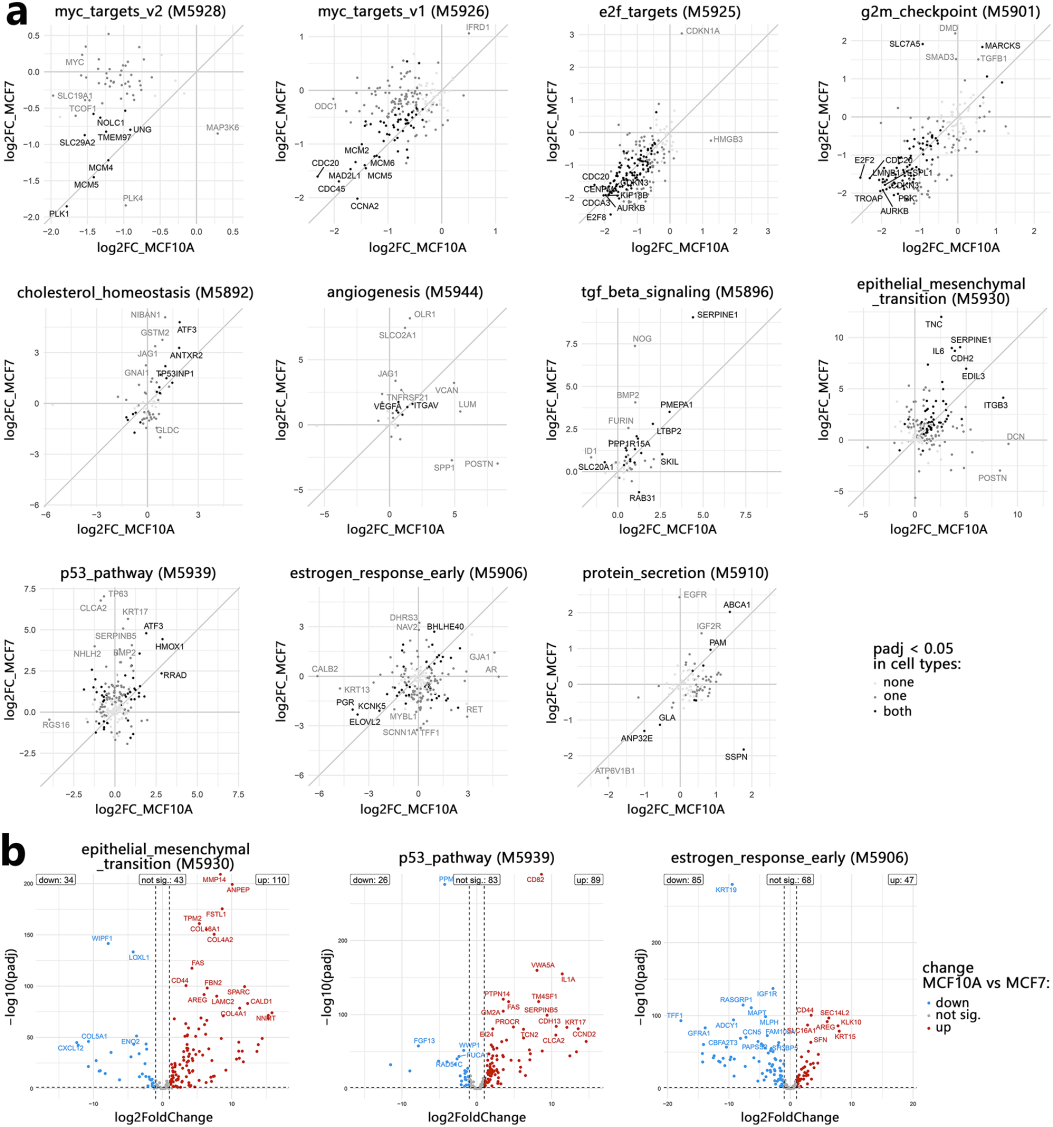
Differences in gene expression profiles between TGFB1-stimulated MCF10A and MCF7 cells. (**a**) Scatterplots of log2-fold changes on the sixth day of TGFB1 stimulation (vs. Ctr) in the genes associated with selected terms from the hallmark collection in MCF10A (X-axis) and MCF7 (Y-axis) cells. (**b**) Volcano plots of the RNA-seq results showing the differentially expressed genes in untreated cells (red color/up – higher in MCF10A cells, blue color/down – higher in MCF7 cells). Each dot represents one gene. Genes with the most significant differences are labeled

More detailed gene set enrichment analyses (GSEAs) based on the whole transcript dataset revealed enrichment in the tgf_beta_signaling (M5896) and, partially overlapping, epithelial_mesenchymal_transition (M5930) pathways from the hallmark collection in both cell lines after both TGFB1 (TGF) and conditioned media (CM) treatment (mainly upregulation) (Fig. 1d; Supplementary data 3). The lack of significant differences between TGF- or CM-treated MCF10A cells (TGF vs. CM) indicates a similar response to both treatments. Minor differences between both treatments in MCF7 cells likely result from weaker responses in CM-treated cells. Comparisons of responses to TGFB1 in MCF10A and MCF7 cells (MCF10A vs. MCF7) revealed differences even in signaling pathways enriched in both cell lines; for example, the tgf_beta_signaling (M5896), epithelial_mesenchymal_transition (M5930), p53_pathway (M5939), and estrogen_response_early (M5906) (Fig. 1d). However, GSEA revealed no significant differences in terms reflecting cell proliferation ability, i.e., e2f_targets (M5925) and, partially overlapping, g2m_checkpoint (M5901) (with apparent inhibition of gene expression). Nonetheless, genes regulated by MYC (especially a subgroup associated with myc_targets_v2) were inhibited more effectively after treatment in MCF10A cells (Fig. 1d). Moreover, greater enrichment in cholesterol_homeostasis (M5892) and angiogenesis (5944) was observed in TGFB1-treated MCF7 cells, while protein_secretion (M5910) was more enriched in MCF10A cells. The comparison of TGFB1-induced changes after 6 days of treatment shown in the scatterplots (Fig. 2a) further illustrates the differences and similarities between cell lines in the response of individual genes associated with the identified signaling pathways. Notably, however, even in pathways similarly enriched in both cell lines, individual genes responded differently (e.g., *CDKN1A*, and *HMGB3* from the e2f_targets pathway or *SMAD3*, *SLC7A5*, and *DMD* from g2m_checkpoint). On the other hand, even in pathways differentially enriched in both cell lines, individual genes responded similarly (e.g., *MCM2*, *MCM4*, *MCM5*, *MCM6* from myc_targets pathways, encoding components of the minichromosome maintenance protein complex) (Fig. 2a).

Interestingly, we observed large differences between both cell lines at baseline (i.e., without TGFB1 treatment), mainly in the expression of genes associated with epithelial_mesenchymal_transition, p53_pathway, and estrogen_response_early (Fig. 1d). In particular, many genes associated with the first two pathways were expressed at higher levels in untreated MCF10A cells (e.g., *COL16A1*, *COL4A1*, *COL4A2*, encoding collagens, or *MMP14* encoding the matrix metalloproteinase), while those associated with the estrogen_response_early were expressed at higher levels in untreated MCF7 cells (Fig. 2b), consistent with their estrogen receptor status. Consequently, SMAD3 and ESR1 were identified as upstream regulators of differentially expressed genes in MCF10A and MCF7 cells, respectively (Figure S4b). Additionally, genes associated with the tgf_beta signaling pathway were differentially expressed in both cell types (Fig. 1d), as shown in detail on the map from the KEGG database (Figure S5a).

### Differences in the TGFβ signaling pathway between MCF10A and MCF7 cells

**Fig. 3:**
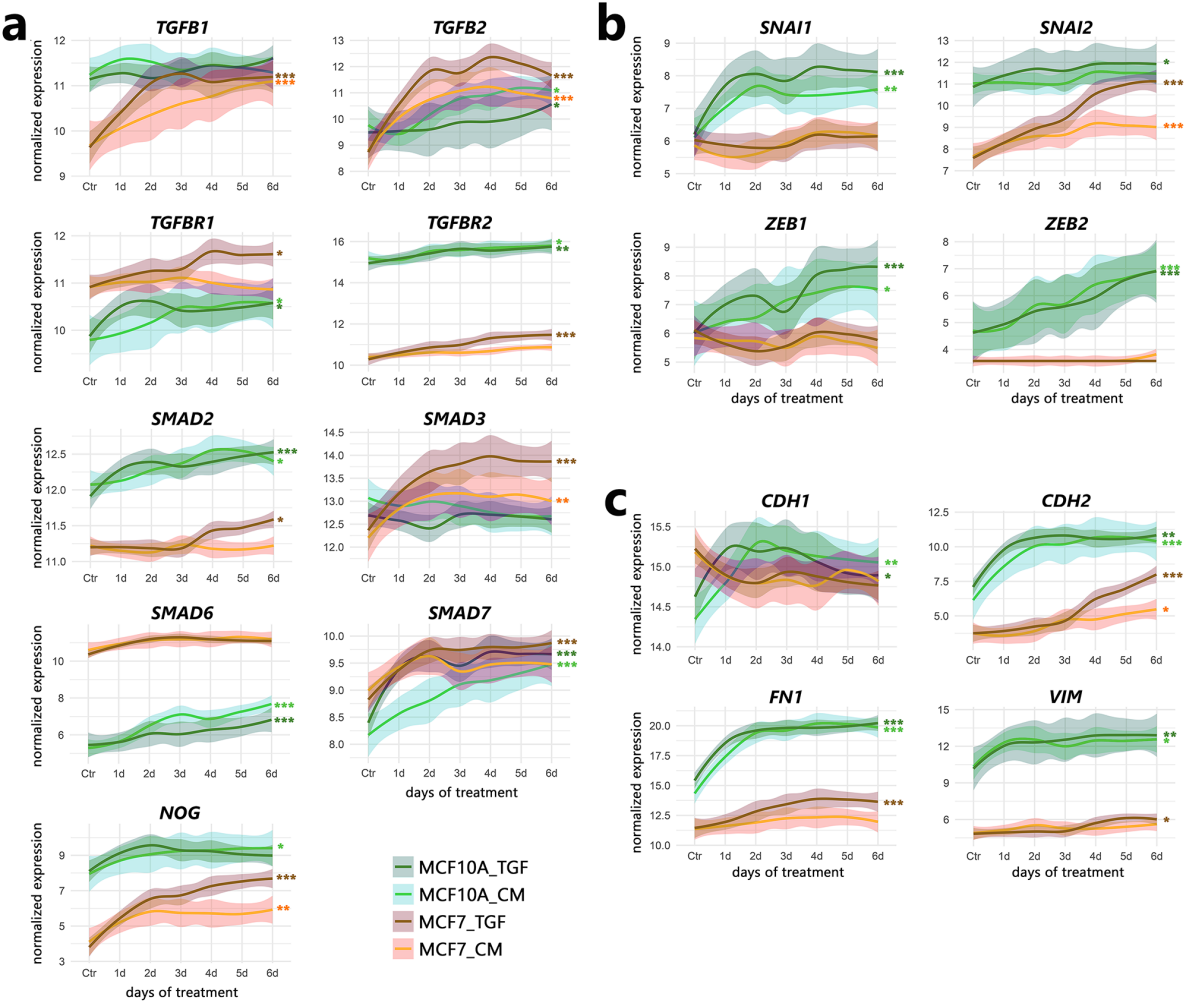
Time trends in the expression of genes related to TGFβ signaling in MCF10A and MCF7 cells. (**a**) Genes involved in SMAD-mediated signaling. (**b**) Transcription factors involved in EMT. (**c**) Epithelial (*CDH1*) and mesenchymal markers. The cells were treated according to the scheme shown in Figure S2a. TGF, direct TGFB1 treatment; CM, conditioned medium treatment. *** padj < 0.0001, ** padj < 0.001, * padj < 0.05 (significance of differences was marked for the entire run if the adjusted p value vs. Ctr reached a given value at least at one experimental point). See also Figure S5b

Detailed analysis of the expression of genes involved in EMT and TGFβ signaling revealed that many genes (e.g., *TGFB1*, *TGFBR2*, *SMAD2*, *NOG*, *SNAI2*, *ZEB2*, *CDH2*, *FN1*, and *VIM*) were initially (Ctr, day 0) expressed at higher levels in MCF10A cells than in MCF7 cells (Fig. 3). Based on this finding, we assumed that TGFB1 signaling through TGFBR2 (TGFβ receptor type II), SMAD2 (receptor-regulated SMAD), SNAI2, and ZEB2 may be more important for maintaining the fibrocystic phenotype (manifested by higher expression of CDH2, FN1, and VIM) of the MCF10A cell line than the cancerous phenotype of MCF7 cells. Genes involved in EMT and TGFβ signaling also responded differently to TGFB1 stimulation in both cell lines (Figs. 2a, S5b). *TGFB1* and *SMAD3* transcript levels significantly increased (reaching a maximum on days 2–3 of treatment) only in MCF7 cells while they did not change substantially in MCF10A cells (Fig. 3a). Therefore, we postulate that in MCF7 cells, TGFB1 signaling is more likely to be prolonged through SMAD3 than SMAD2. Consistent with this finding, phosphorylated/active SMAD3 was more efficiently stabilized in MCF7 cells (Figures S1c, d, and 5b). In parallel with the activation of R-SMADs, factors that deactivate the pathway can be upregulated after TGFB1 treatment. These are SMAD6 and SMAD7; i.e., inhibitory SMADs, or NOG, a potent inhibitor of BMP (bone morphogenetic protein) cytokines, which are known to trigger signaling through SMADs (similar to TGFβ but via SMAD1, SMAD5, or SMAD8). Indeed, we observed *SMAD6*, *SMAD7*, and *NOG* upregulation (and only *SMAD6* transcript levels in MCF7 cells, initially high, did not change). However, it is noteworthy that *SMAD6* and *SMAD7* (but not *NOG*) transcript levels were considerably lower in untreated MCF10A cells (Fig. 3a). This presumably facilitates the maintenance of higher signaling activity through R-SMADs in these cells, resulting in the enrichment of the epithelial_mesenchymal_transition pathway (110 of 200 genes from the hallmark collection were expressed at higher levels in MCF10A cells than in MCF7 cells; Fig. 2b). The TGFβ signaling can also be inactivated/modulated by SMAD-specific E3 ubiquitin protein ligases (SMURF1 and SMURF2, whose transcript levels increased more strongly after TGFB1 treatment in MCF10A cells) and latent-transforming growth factor beta-binding proteins (LTBP1, LTBP2, LTBP3), furin (FURIN), and decorin (DCN), whose transcript levels were differentially regulated after TGFB1 treatment in both cell lines (Figure S5b, Supplementary data 1).

Similar changes in the expression of genes involved in TGFβ signaling were observed in cells directly stimulated with TGFB1 and those treated with CM (although the response was frequently delayed or weaker in the second scenario), indicating the secretion of TGFβ pathway mediators, including TGFB1 itself in the case of MCF7 cells (see Figure S2b), into the media. Genes encoding other potential regulators of the TGFβ signaling pathway, including *TGFB2*, *BMP2*, *BMP4*, *BMP6*, *BMP7* (BMP cytokines), *INHBA*, *INHBB*, *INHBE* (activins and inhibins), or *GDF11*, *GDF15* (growth differentiation factors) and the receptors *BMPR1B*, *BMPR2*, *ACVR1*, and *ACVR1C*, were upregulated in at least one cell line (Figure S5b and Supplementary data 1).

Among the transcription factors involved in the later stages of TGFβ and EMT signaling, we observed a several-fold increase in *SNAI1* (as well as *ZEB1* and *ZEB2*) transcript levels starting from the first day of treatment only in MCF10A cells (stimulated by either TGFB1 or CM), while *SNAI2* was more effectively upregulated in MCF7 cells (but its transcript was still at a lower level than that in MCF10A cells) (Fig. 3b). Surprisingly, increased expression of *SNAI1* and *SNAI2* was accompanied by an increase (albeit small) rather than the expected decrease in *CDH1* transcript levels in MCF10A cells, while the decrease in *CDH1* levels was not significant in MCF7 cells (Fig. 3c). However, the expression of mesenchymal markers, *CDH2*, *VIM*, and *FN1*, was induced to some extent in both cell lines. Nevertheless, western blot analysis showed that the CDH1 protein levels decreased in both cell lines after TGFB1 treatment and confirmed the accumulation of VIM in MCF10A cells (Fig. 5b), reflecting its expression at the mRNA level. In conclusion, although the TGFβ and EMT signaling pathways were enriched in both cell lines starting from the first day of treatment with TGFB1 or conditioned media, there were considerable differences between the cell lines (partially resulting from differences existing before treatments).

### TGFB1 treatment affects the expression of genes associated with cell cycle progression

**Fig. 4:**
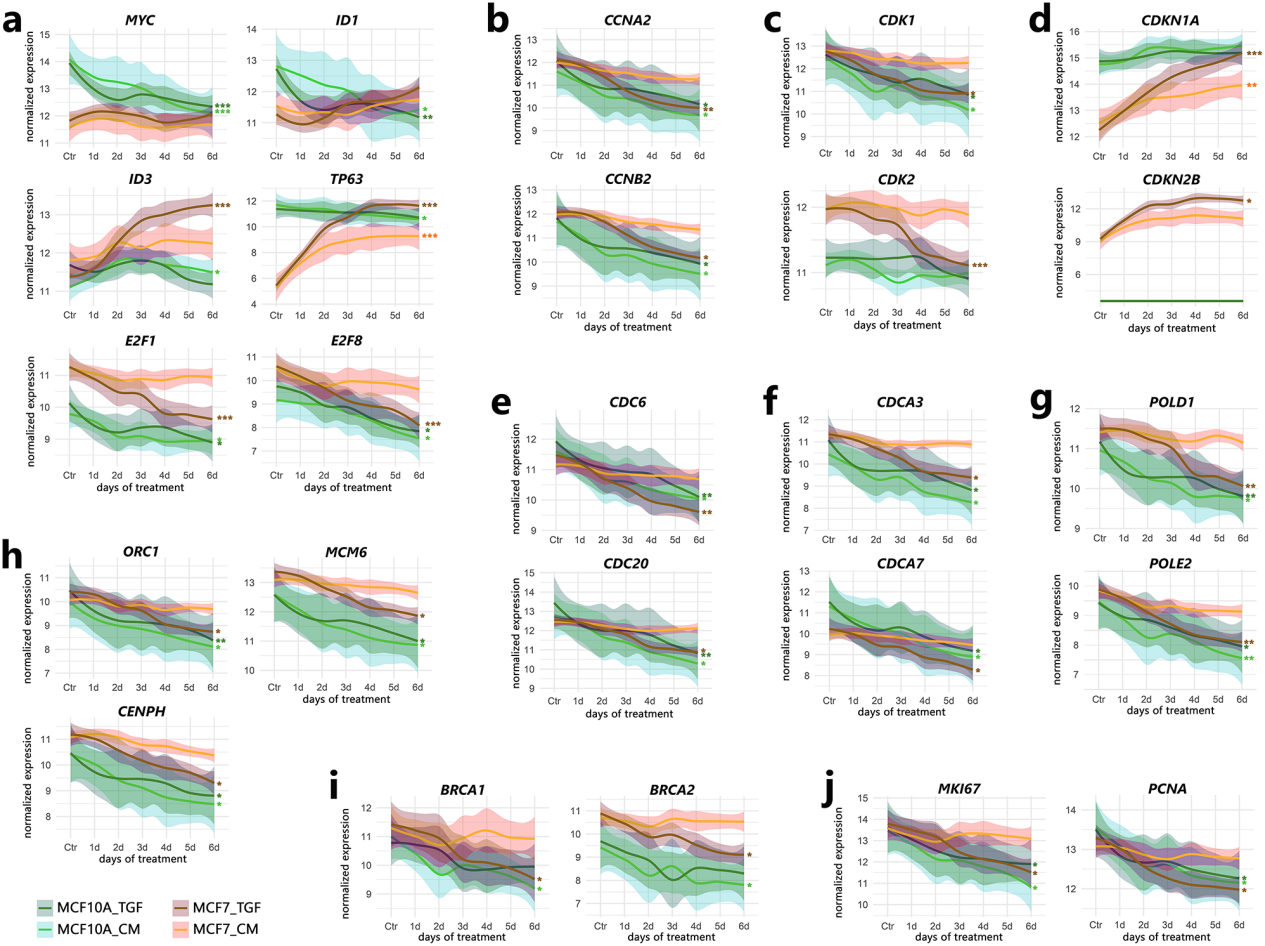
Time trends in the expression of genes related to cell cycle progression in MCF10A and MCF7 cells. Examples of (**a**) transcriptional regulators, (**b**) cyclins, (**c**) cyclin-dependent kinases and (**d**) their inhibitors, (**e**) cell division cycle proteins, (**f**) cell division cycle associated proteins, (**g**) DNA polymerases, (**h**) proteins involved in chromosomal replication, (**i**) proteins involved in DNA repair and (**j**) markers of proliferation. The cells were treated according to the scheme shown in Figure S2a. TGF, direct TGFB1 treatment; CM, conditioned medium treatment. *** padj < 0.0001, ** padj < 0.001, * padj < 0.05 (significance of differences was marked for the entire run if the adjusted p value vs. Ctr reached a given value at least at one experimental point). See also Figures S6 (cell cycle in KEGG pathways) and S7 (time trends of other cell cycle-related genes)

In addition to inducing EMT, TGFβ is known to inhibit cell-cycle progression by blocking the G1 phase resulting from decreased expression of the MYC proto-oncogene [35] and IDs (inhibitors of DNA binding) [36]. Accordingly, *MYC* and *ID1* expression was inhibited, but only in MCF10A cells (Fig. 4a). Consequently, MYC targets (M5926 and M5928 from the hallmark collection) were also largely downregulated mainly in MCF10A cells (Figs. 1d and 2a). In contrast, the expression of *ID3* was upregulated (more clearly in MCF7 cells; Figure S7a). Nevertheless, the downregulation of genes involved in cell-cycle progression, both during the G1/S transition (e2f_targets, M5925) and during the G2/M transition (g2m_checkpoint, M5901), was also observed in MCF7 cells (Fig. 1d; see also Figure S6a). However, our data revealed that cell-cycle inhibition could be achieved by different mechanisms, such as MYC inhibition in MCF10A cells and a p53-dependent mechanism in MCF7 cells (Figs. 1d and 2a, and S6b). The strong upregulation of *TP63* (according to ChEA3, it may be responsible for the upregulation of ~ 9% of genes in MCF7 cells; Figure S4b) and cyclin-dependent kinase (CDK) inhibitors: *CDKN1A* (encoding p21) and *CDKN2B* (encoding p15) was observed specifically in MCF7 cells (Fig. 4a, d; although some other *CDKN* genes, e.g., *CDKN2C*, were downregulated; Figure S7b).

**Fig. 5:**
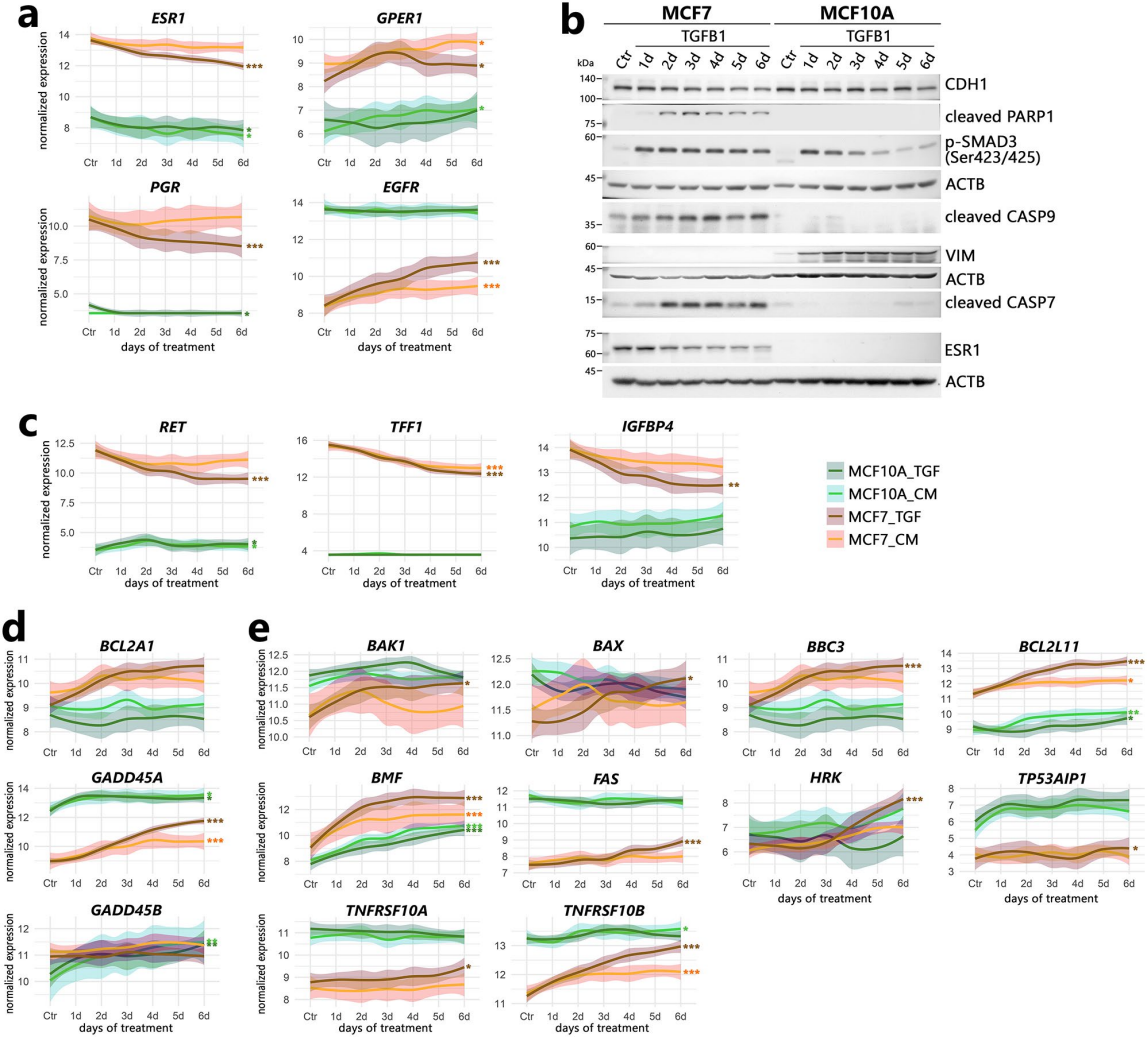
Inhibition of estrogen signaling and induction of cell death after TGFB1 treatment in MCF7 cells. Time trends in the expression of genes related to estrogen signaling and apoptosis in MCF10A and MCF7 cells: (**a**) steroid receptors and *EGFR*; (**c**) ESR1 targets; (**d**) prosurvival genes; (**e**) proapoptotic genes. The cells were treated according to the scheme shown in Figure S2a. TGF, direct TGFB1 treatment; CM, conditioned medium treatment. *** padj < 0.0001, ** padj < 0.001, * padj < 0.05 (significance of differences was marked for the entire run if the adjusted p value vs. Ctr reached a given value at least at one experimental point). (**b**) Response of MCF7 and MCF10A cells to TGFB1 treatment analyzed by Western blot. The cells were treated according to the scheme shown in Figure S2a. The levels of the indicated proteins were analyzed in total protein extracts. ACTB was used as a loading control

Therefore, growth arrest and DNA damage (*GADD45A* and *GADD45B*) genes were upregulated (Fig. 5d), while genes encoding activators of cell-cycle progression were downregulated (Figure S6a) after TGFB1 treatment in both cell lines. This phenomenon applies to genes encoding several family members of the E2F transcription factors, namely, *E2F1*, *E2F2*, *E2F4*, and *E2F8* (Figs. 4a, S7a), their dimerization partner *TFDP1*, and the pocket protein *RBL1*; but not *RB1* and *RBL2* (Figure S7a), cyclins and CDKs, i.e., G1-specific *CCNE2*, *CCNA2* and its targets: *CDK2* during S phase, and *CDK1* during the transition from G2 to M, G2/M-specific: *CCNB1*, *CCNB2*, *CCNF*, but not cyclins D (Figs. 4b, c, S7b), cell division cycle proteins: *CDC6*, *CDC7*, *CDC20*, *CDC25A*, *CDC25C*, *CDC45* (Figs. 4e, S7c), and cell-division cycle associated proteins, such as *CDCA2*, *CDCA3*, *CDCA5*, *CDCA7*, *CDCA7L*, and *CDCA8* (Figs. 4f, S7d). Furthermore, some genes encoding DNA polymerases, e.g., *POLA1*, *POLA2*, *POLD1*, *POLD2*, *POLE*, and *POLE2* (Figs. 4g, S7e), and proteins involved in chromosomal replication, e.g., origin recognition complex subunits, *ORC1* and *ORC6*, as well as all minichromosome maintenance complex components, i.e., *MCM2*-*7*, centromere proteins, *CENPA*, *CENPE*, *CENPF*, *CENPH*, *CENPM*, and others (Figs. 4h, S7f), were downregulated in both cell lines. Interestingly, some genes encoding proteins involved in DNA repair (*BRCA1*, *BRCA2*, *DCLRE1A*, *DCLRE1B*, *ERCC6L*, and *MRE11*) were more effectively inhibited in MCF7 cells (Figs. 4i, S7g). Consequently, the marker of proliferation *MKI67* and the proliferating cell nuclear antigen *PCNA* were downregulated in both cell lines (Fig. 4j).

### Inhibition of estrogen signaling and induction of cell death after TGFB1 treatment in MCF7 cells

Treatment with TGFB1 affected genes related to estrogen signaling, which is essential for maintaining the phenotype of MCF7 cells. Specifically, many members of the estrogen_response_early (hallmark M5906) were downregulated in the MCF7 cell line (Fig. 1d). This may be due to the downregulation of the estrogen receptor *ESR1*, both at the mRNA and protein levels (Fig. 5a, b), which correlated with decreased expression of several direct ESR1 targets (known to be stimulated by estrogen; [37]), e.g., *RET*, *TFF1*, and *IGFBP4* (Fig. 5c). It is noteworthy that transcription factor enrichment analysis (using ChEA3) identified ESR1 as a regulator of ~ 10% of all TGFB1-downregulated genes (98 out of 975) in MCF7 cells (while downregulated E2F family members were identified as transcriptional regulators in both cell lines; Figure S4b). This indicates that reducing ESR1 expression may have a large impact on the expression profile and may contribute to the inhibition of proliferation in TGFB1-treated MCF7 cells. TGFB1 treatment also inhibited the expression of *PGR* (progesterone receptor), while *GPER1* (G-protein coupled estrogen receptor) was slightly upregulated (Fig. 5a). TGFB1 can also deregulate various signaling pathways (NOTCH, WNT/β-catenin, PI3K/AKT, EGFR, RAS/RAF/MAPK) known to stimulate cell growth, survival and differentiation in other molecular subtypes of breast cancer (Figure S8; specifically, the activation of epidermal growth factor receptor, *EGFR*, expression in MCF7 cells is of interest; Fig. 5a).

The induction of cell death in MCF7 cells was the most important functional difference between MCF7 and MCF10A cells in response to TGFB1. This could be executed by caspases 7 and 9 (caspase 3 is not expressed in MCF7 cells due to a mutation leading to a frameshift in the *CASP3* gene). The accumulation of active forms of caspases correlated with PARP1 cleavage (Fig. 5b) and indicated the induction of caspase-dependent apoptosis. Analysis of the apoptosis network (KEGG ID: hsa04210; Figure S9) revealed upregulation of some prosurvival genes in one cell line or the other (e.g. *BCL2A1*/A1, *GADD45A*, and *GADD45B*; Fig. 5d). On the other hand, the expression of proapoptotic genes: *BAK1* (*BAK*), *BAX*, *BBC3* (*PUMA*), *BCL2L11* (*BIM*), *FAS*, *HRK*, *TP53AIP1* (*p53AIP1*), *TNFRSF10A* and *TNFRSF10B* (*TRAILR2*) was preferentially induced in MCF7 cells (only *BMF* was similarly induced in both cell lines; Figs. 5e and S9). We assumed that apoptotic MCF7 cells had reduced secretory functions (hallmark protein_secretion collection was enriched only in MCF10A cells; Fig. 1d), which resulted in a weak propagation of TGFB1 signaling through conditioned media (particularly in terms related to cell-cycle progression; Fig. 4).

The observed proapoptotic and antiestrogenic effects of TGFB1 treatment in MCF7 cells may suggest the prognostic value of *TGFB1* expression in breast cancer patients. Interestingly, the Kaplan Meier analysis of a large cohort of breast cancer patients (*n* = 2,976) integrated by Győrffy [38] (available at https://kmplot.com/analysis/index.php?p=service&cancer=breast_rnaseq_gse96058) revealed that high *TGFB1* levels are associated with a better prognosis, but only in luminal A patients (Figure S10). These tumors are characterized by the presence of ER and/or PR, the absence of HER2, and low expression of Ki-67.

## Discussion

The cytostatic effect, one of an essential functions of TGFβ signaling, is explained by several molecular mechanisms that depend on the cellular context [39]. Our results indicate that TGFB1 inhibits proliferation in both MCF10A and MCF7 breast epithelial cell lines through different mechanisms. Repression of *MYC* expression, which is a central event in the TGFB1 cytostatic response, was observed only in noncancerous MCF10A cells. Cells that fail to downregulate MYC expression are usually resistant to growth inhibition mediated by TGFB1 [35]. Nevertheless, MCF7 cells also responded through the upregulation of cell-cycle inhibitors, especially *CDKN1A* (encoding p21) and *CDKN2B* (encoding p15). The transcription of both genes has been shown to be upregulated in response to TGFβ [39–43]. These CDK inhibitors can arrest the cell cycle in the early G1 phase. In particular, CDKN2B interacts strongly with CDK4 and CDK6, which are important for cell-cycle G1 phase progression, while CDKN1A potentiates CDK2 inhibition [42–44]. The *CDKN1A* gene can be transcriptionally activated by p53 [45]. Interestingly, however, the induction of *CDKN1A* by TGFβ is not dependent on wild-type p53 [40, 41], which is present in MCF7 cells. We postulate that *CDKN1A* can be activated by another member of the p53 family, p63, whose transcription is strongly upregulated after treatment with TGFB1 and CM only in MCF7 cells. To date, at least some p63 isoforms have been shown to upregulate archetypal p53 target genes (including *CDKN1A*) and induce cell-cycle arrest and apoptosis [46]. However, both *TP63* and *CDKN1A* are persistently expressed at high levels in MCF10A cells. Thus, CDKN2B (p15), which is not expressed in MCF10A cells, could be responsible for the antiproliferative effect of TGFβ specifically in MCF7 cells. It is worth noting that TGFB1-induced cell cycle arrest mediated by p15 and p21 can also induce cellular senescence [47, 48], which was seen in a fraction of MCF7 cells.

TGFβ acts as a potent inhibitor of proliferation and inducer of apoptosis in the early stages of breast cancer but promotes cancer aggressiveness through the induction of EMT and suppression of antitumor immune responses in advanced stages. The dual effects of TGFβ on cancer development are known as the TGFβ paradox [15, 16]. We found that in MCF7 cells, TGFB1 acts as an inhibitor of proliferation and inducer of cell death (possibly apoptosis), which suggests that these cells originated from early-stage cancer. Indeed, MCF7 cells are being used as a model for luminal type-A breast cancer, which tends to grow slowly and is less aggressive than other breast cancer types. However, these cells have been isolated from pleural effusion and thus from metastatic cancer [49]. In our experimental model, TGFB1 induced changes (downregulation of the epithelial marker CDH1 and upregulation of several mesenchymal markers) that could enable EMT in a fraction of MCF7 cells (which is linked to the formation of metastases). However, mechanisms leading to apoptotic death are induced in a subset of these cells, which is illustrated by the activation of caspase 7 and caspase 9 (caspase 3 is absent in MCF7 cells), as well as PARP1 cleavage. TGFβ-induced apoptosis is well documented in many cell types (lymphocytes and hepatocytes are particularly sensitive). This response is cell type- and context-dependent; thus multiple apoptotic mediators and signaling pathways leading to TGFβ-induced death have been proposed. Most mediators appear to target the mitochondrial (intrinsic) pathway, which involves BCL-2 family members [50]. BCL2L11 (Bcl-2-like protein 11) and BMF (Bcl-2-modifying factor) have been found to play central roles in SMAD3/SMAD4-dependent TGFβ-mediated apoptosis, as well as in normal mouse mammary epithelial cells [51]. We also found upregulation of several proapoptotic Bcl-2 family members (*BCL2L11*, *BAK1*, *BAX*, and *BBC3*) after TGFB1 stimulation, specifically in MCF7 cells. In contrast, transcription of the *BMF* gene was upregulated in both the MCF7 and MCF10A cell lines, suggesting that this factor is not sufficient to promote apoptosis. However, among the proapoptotic genes specifically upregulated in MCF7 cells was also *TNFRSF10B* (tumor necrosis factor receptor superfamily member 10B, also known as *DR5*, death receptor 5, or *TRAILR2*, TRAIL receptor 2), which suggested the involvement of the extrinsic pathway (initiated by death receptor signaling) in TGFβ-mediated apoptosis. It should be noted that apoptosis is an important process during breast remodeling after postlactational involution and can be regulated by TGFβ [52]. This effect may be mediated by SMAD4, whose overexpression induces BIM and BAX, followed by apoptosis in ER-positive breast cancer cells [53]. Thus, we can speculate that in the mammary gland, in the presence of high levels of TGFβ, only ER-positive cells are eliminated.

The most studied function of TGFβ is the activation of EMT. When we compared transcriptional changes in cancerous MCF7 cells and noncancerous MCF10A cells, it was evident that TGFB1 induced EMT in both cell lines, but slightly different mechanisms may have been involved. The MCF10A cell line has been frequently used as a model in genomic studies of TGFβ signaling and EMT, yet different underlying mechanisms have been proposed. One assumed that TGFB1-induced EMT in these cells involves two double-negative feedback loops: one between the transcription factor SNAIL1 and the miR-34 family and another between the transcription factor ZEB1 and the miR-200 family [54]. However, other study has indicated that the SNAIL and ZEB1 proteins have little effect, while JUNB (the AP1 subunit) is anticipated to have a much greater effect on TGFB1-induced EMT [55]. Based on single-cell analysis of MCF10A cells, the NOTCH signaling pathway has been proposed to be another key driver of TGFβ-induced EMT [56]. To date, the most comprehensive resource of information on TGFβ and EMT signaling in MCF10A cells is the work of the Emili group [57]. They quantified more than 61,000 molecules from 12 omics and, among others, identified four distinct cell states during EMT. Not surprisingly, the integration of the transcriptome and various proteomic layers has highlighted the pitfalls of assigning gene functions based on a single omics approach, and revealed fundamental differences in regulatory mechanisms operating at different levels. Nevertheless, changes in the expression of nearly 36% of genes (1,426) determined at the transcriptional level correlated well with changes at the protein level [57]. This group included many EMT-related factors. In our study, we analyzed the changes in the transcriptome, which limited its impact. However, our main goal here was to compare two different scenarios (direct and indirect, i.e. mediated by conditioned media) of TGFB1 action in two cell lines representative of noncancerous and cancerous epithelium. We found that the baseline expression of many genes related to EMT was much greater in MCF10A cells than in MCF7 cells. Thus, we concluded that MCF10A cells may exhibit mesenchymal-like phenotypes even without TGFB1 treatment. Indeed, it has been estimated that MCF10A cells with a mesenchymal or hybrid epithelial–mesenchymal phenotype can make up as much as 50–74% of the population, depending on growth conditions [58]. This phenomenon could be explained by the origin of the MCF10A cells, which were isolated from the mammary gland of a patient with fibrocystic disease [59]. Therefore, the use of MCF10A cells as a model of “normal” mammary gland epithelium has already been questioned [60]. Our results also raise the question of whether MCF10A cells are the best model for studying TGFβ-induced EMT mechanisms in “normal” epithelium. Interestingly, our results indicate that TGFB1-induced signaling does not lead to the increased release of TGFB1 into the culture media in MCF10A cells, so the signal must be propagated by other mediators.

It is worth mentioning that EMT in ER-positive MCF7 cells can also be induced by the loss of ESR1 and subsequent activation of the EGFR–ERK signaling pathway [61]. Thus, the inhibition of *ESR1* expression and upregulation of *EGFR* observed after TGFB1 treatment may further support EMT and metastasis. Indeed, decreased ESR1 expression can promote the metastasis of ER-positive breast cancer [62]. TGFβ can also reduce the activation of ESR1 after estrogen treatment (thus inhibiting its mitogenic action) and may be a driver of bone metastases [63]. TGFβ-induced EGFR upregulation in breast cancer cells can be mediated by the canonical SMAD3 and ERK/SP1 signaling pathways [64]. Moreover, EGFR activity is required for TGFβ-induced invasion and migration in breast cancer cells [65]. EGFR can cooperate with TGFBR1 via the MEK/ERK pathway, enabling the induction of AP1 (JUN/FOS). In addition, p63 is crucial for this signaling. Indeed, ChEA3 analysis revealed that AP1 family members and p63 are important transcription factors involved in gene activation in TGFB1-treated MCF7 cells. On the other hand, some reports have shown that ESR1 can inhibit TGFβ signaling by inducing the degradation of SMAD in an estrogen-dependent manner through AP1 [66–69]. Thus, TGFβ and ER signaling reciprocally suppress each other.

Our results indicate that TGFB1-induced SMAD-mediated signaling is different in MCF10A and MCF7 cells. In addition to the differences in the mRNA expression levels of TGFβ receptors and *SMAD2* in untreated cells, it is worth noting that *SMAD3* is upregulated only in MCF7 cells. Moreover, after reaching a maximum of approximately one hour of treatment, SMAD3 phosphorylation/activity was very rapidly extinguished in MCF10A cells but not in MCF7 cells. These findings suggest that TGFB1 signaling is more likely to be prolonged through SMAD3 than through SMAD2 signaling in MCF7 cells. SMAD2 and SMAD3 are known to form hetero-oligomeric (heterodimers or heterotrimers) complexes with SMAD4 and translocate to the nucleus [70, 71]. Different SMAD complexes have different impacts on the transcriptional response [72]. SMAD2/SMAD4 complexes do not bind DNA alone but require other transcription factors to target them to specific sequences [73]. This may explain why SMAD2 was not identified as a regulator of TGFβ-stimulated genes in the ChEA3 analysis (we used the ReMap library based on ChIP-seq experiments). SMAD3/SMAD4 complexes can bind directly to SMAD-binding elements in the promoters of target genes but synergize with other transcription factors to regulate gene expression [73]. Thus, not only the stoichiometry of SMAD complexes but also the whole composition of the SMAD interactome determines their activity. It is worth noting that SMAD3 has the V134F mutation (in the central linker region) in MCF7 cells. This raises the question of to what extent this SMAD3 mutation determines differences in TGFβ signaling between cell lines (e.g., affecting the stability and/or interactome of this protein).

An intriguing observation of our study was that the responses of both cell lines (MCF7 and MCF10A) to direct treatment with TGFB1 and exposure to conditioned media from TGFB1-treated cells were quite similar (as suggested by GSEA). This finding indicated that TGFB1 signaling can be further extended as a bystander effect mediated by a large quantity of secretory molecules, such as cytokines, activins and inhibins, or growth factors, including TGFβ itself. It is known that the secretion of active forms of TGFB1 and TGFB2 from MCF7 cells is increased, for example, under treatment with antiestrogens [74], which are often used to treat ER-positive breast cancer. In addition, TGFβ is released following the exposure of cells to ionizing radiation [75]. Therefore, TGFβ-induced bystander effects may have implications for cancer progression and therapy.

Secreted TGFβ can further induce a response not only in cancer cells but also in the tumor microenvironment. Multiple autocrine loops maintain the secretion of prometastatic factors, especially by cancer-associated fibroblasts [76]. As a result, TGFB1 can promote targeted migration of breast cancer cells through the lymphatic system [77], inhibit lymphatic growth and promote vascular angiogenesis [78], or contribute to resistance in endocrine-related cancers [79]. In addition, within the tumor microenvironment, TGFβ is the most potent suppressor of immune system activity against cancer cells. In normal tissues, TGFβ triggers the expression of prooxidant and profibrotic genes, leading to fibrosis, genomic instability, and other side effects. In this regard, research on TGFβ-targeted drugs that could be combined with other anticancer treatments is underway [80]. One key aspect remains the identification of reliable biomarkers for TGFβ-targeted treatment. High TGFB1 protein levels in tumor tissue were associated with poor prognosis in breast cancer patients, at least in some reports [81]. On the other hand, the evaluation of TGFβ in the serum/plasma of breast cancer patients did not reveal the prognostic value of circulating TGFβ [82, 83]; however, the small sample size was a limitation of these reports. Our results indicate that high TGFβ may be beneficial only for a subset of breast cancer patients (with estrogen receptor expression). Nevertheless, although TGFβ induces apoptosis and inhibits the mitogenic effect of the ER, some cells are stimulated by TGFβ to change their phenotype to mesenchymal, which can promote metastasis.

## Conclusions

Transcriptome profiling revealed that stimulation of noncancerous (MCF10A) and cancerous (MCF7) epithelial cells with TGFB1 cytokine resulted not only in the activation of genes related to extracellular matrix organization and the EMT process but changes in the expression of genes related to cell-cycle progression and DNA replication. Each cell line showed a slightly different response to TGFB1 stimulation, resulting from the activation of different pathways. The inhibition of proliferation by downregulation of *MYC* transcription was typical only for MCF10A cells. In contrast, genes associated with activation of the p53 signaling pathway (primarily *TP63*,* CDKN1A*, and *CDKN2B*) were more strongly stimulated in MCF7 cells. The most pronounced differences between the cell lines included the induction of cell death (via the activation of caspase 7 and caspase 9 and PARP1 cleavage) and the inhibition of the estrogen response signaling pathway in the estrogen receptor-positive MCF7 cancer cell line. Considering that TGFβ tends to induce apoptosis at early stages of cancer development and that the MCF7 cell line was isolated from a pleural effusion (and therefore from a metastatic site, indicative of later stages of carcinogenesis), this was an unexpected result. It is possible, however, that after metastasis, the tumor cells underwent further changes that sensitized them to TGFβ-induced apoptosis. Since these cells have an epithelial phenotype, it can be assumed that two processes occur during metastasis – EMT and reversible MET. Alternatively, metastasis of cells with an epithelial phenotype was possible with the assistance of other cells. We postulate that estrogen receptor-positive breast cancer patients may benefit from high levels of *TGFB1* expression due to the repression of estrogen receptor signaling, inhibition of proliferation, and induction of apoptosis in cancer cells. However, some cells may undergo EMT, which increases the risk of metastasis.

The results of our study revealed similar changes in gene expression profiles in cells directly stimulated with TGFB1 and in cells exposed to factors released into the culture medium by TGFB1-stimulated cells (conditioned medium). This indicates the presence of a TGFβ-induced bystander effect and suggests that the effect induced in cells by TGFβ may be transmitted to naïve cells that were not directly exposed to this cytokine. This phenomenon may extend the duration of TGFβ-induced signaling and lead to the activation of TGFβ-dependent pathways in the tumor microenvironment that are not directly exposed to this cytokine.

## Electronic supplementary material

Below is the link to the electronic supplementary material.

Supplementary Material 1: **Supplementary data 1** RNA-seq analyses of the effect of TGFB1 and conditioned media on the transcriptome in MCF10A and MCF7 cell lines. TGFB1 or CM-induced changes sheet: changes in gene expression relative to untreated cells; base mean expression > 100, log2(FC) > 2 and < − 2, and padj < 0.05 have been color-coded. Ctr_MCF10A vs. MCF7 sheet: comparison of expression levels in untreated cells. Excel document

Supplementary Material 2: **Supplementary data 2** List of genes (with data on expression changes vs. Ctr) in clusters identified by hierarchical clustering analysis based on expression trends (and on preselected RNA-seq data) in MCF10A and MCF7 cells treated with TGFB1. padj < 0.05 have been color-coded. Data related to Fig. 1C. Excel document

Supplementary Material 3: **Supplementary data 3** Gene set enrichment analyses (GSEA) based on RNA-seq data: pathways from the hallmark collection. Data related to Fig. 1C, D. Excel document

Supplementary Material 4: **Supplementary Figures**. PDF document

Supplementary Material 5: **Original blots**

## Data Availability

The datasets generated and analyzed during the current study are available in the Array Express collection repository; acc. E-MTAB-13865 [https://www.ebi.ac.uk/biostudies/arrayexpress/studies/E-MTAB-13865].

## Abbreviations

CDH1: E-cadherin
CDH2: N-cadherin
CDK: Cyclin-dependent kinase
ChEA3: ChIP-X Enrichment Analysis 3
CM: Conditioned medium
Co-SMAD: Common partner SMAD (SMAD4)
EGFR: Epidermal growth factor receptor
EMT: Epithelial–mesenchymal transition
ER: Estrogen receptor
ESR1: Estrogen receptor alpha (ERα)
FET p value: Fisher’s exact test p value
GSEA: Gene set enrichment analysis
I-SMADs: Inhibitory SMADs (SMAD6 and SMAD7)
MET: Mesenchymal–epithelial transition
padj: Adjusted p value
PARP1: Poly [ADP-ribose] polymerase 1
R-SMADs: Receptor-regulated SMADs (SMAD2 and SMAD3)
TGFβ: Transforming growth factor beta (TGFB1, TGFB2, TGFB3)
TGFB1: Transforming growth factor beta 1
