# Supplementary material for: Transcriptional responses to direct and indirect TGFB1 stimulation in cancerous and noncancerous mammary epithelial cells
Source: Cell Commun Signal. 2024 Oct 28;22:522. doi: 10.1186/s12964-024-01821-5 (PMC11514872; doi:10.1186/s12964-024-01821-5)
Supplement: Supplementary file 4 — Supplementary Material 4: Supplementary Figures. PDF document [file 12964_2024_1821_MOESM4_ESM.pdf]

Supplementary Figures

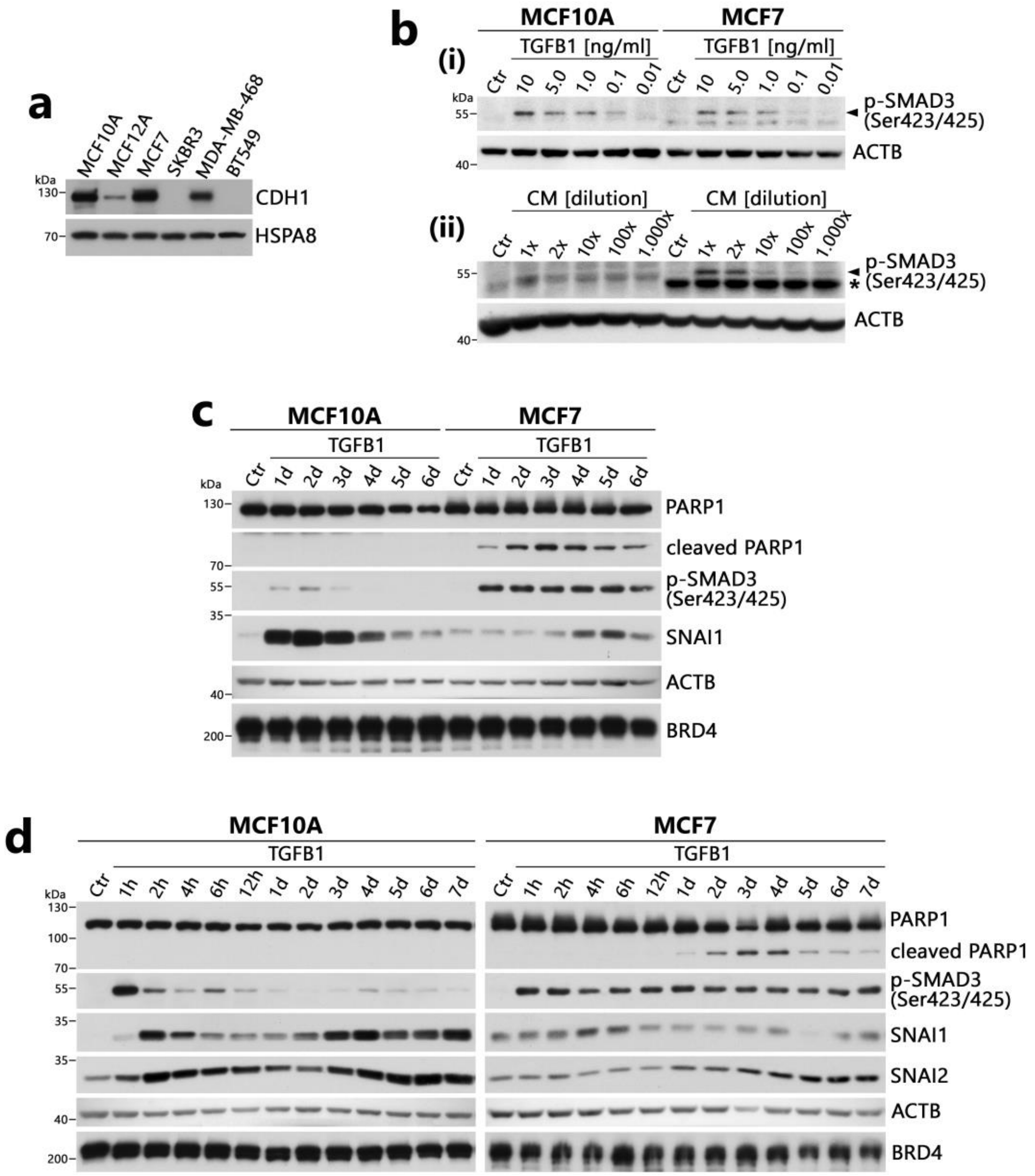

Figure S1. Response of breast epithelial cells to TGFB1 treatment analyzed by Western blot. (**a**) Expression levels of the epithelial marker CDH1 in a panel of breast epithelium-derived cells. (**b**) SMAD3 phosphorylation after one-hour treatment of MCF10A and MCF7 cells by (i) TGFB1 at a concentration ranging from 10 to 0.01 ng/ml or (ii) serial dilutions of conditioned medium (CM) collected 22h after treating cells with 10 ng/ml TGFB1 for 2 hours (pulsed treatment). Unspecific protein band is marked with an asterisk. (**c** and **d**) Response of MCF10A and MCF7 cells to TGFB1 treatment at a concentration of 10 ng/ml. Cells were treated continuously for 6 or 7 days. Starting from day 1, half of the medium was replaced daily with fresh TGFB1. The levels of indicated proteins were analyzed in total protein extracts (a, b) or nuclear extracts (c, d). BRD4 and HSPA8 or ACTB were used as loading controls.

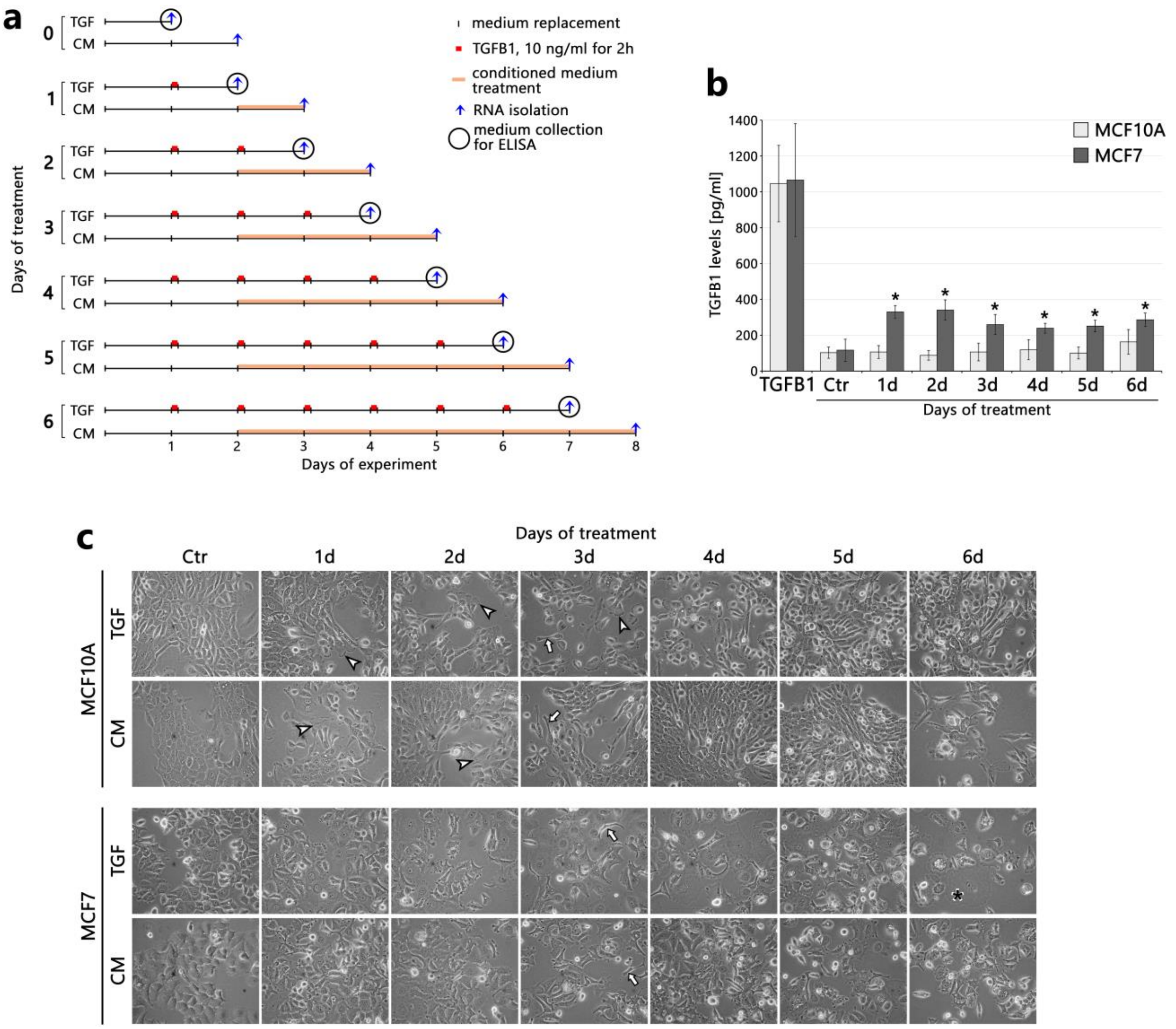

Figure S2. (**a**) Scheme for treating and harvesting cells for RNA-seq analysis. Conditioned media from cells directly treated with TGFB1 for 2 hours (TGF) were collected daily 22 hours after replacing the media with fresh media and administrated to other cells (CM). The cells were seeded in an amount that allowed them to be harvested at ~80% confluence. (**b**) The level of recombinant TGFB1 reconstituted in culture media at calculated concentration of 10 ng/ml and native TGFB1 in conditioned media were analyzed by ELISA. Significance of differences between media collected from TGFB1 treated and untreated cells: *$p < 0.05$. (**c**) Morphology of TGFB1-treated cells. Phase-contrast images (magnification 400x). Examples of polarized cells forming lamellipodia (arrowheads; typical of MCF10A cells), elongated cells (arrows), and a senescent cell (asterisk; typical of MCF7 cells) are shown. The dead, floating cells were removed before the image was taken.

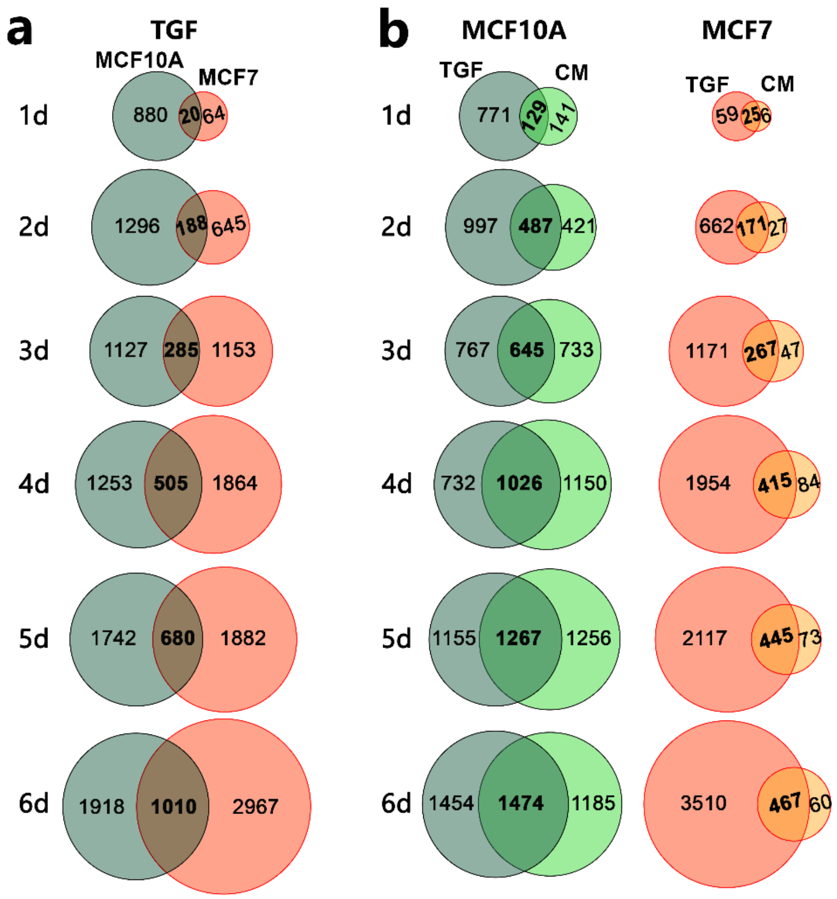

Figure S3. Comparison of global transcriptional changes (RNA-seq analyses) in MCF10A and MCF7 cells after direct TGFB1 (TGF) or conditioned medium (CM) treatment. (**a**) Overlap of genes affected after TGFB1 treatment in both cell lines. (**b**) Overlap of genes affected after TGFB1 (TGF) or conditioned medium (CM) treatment. Cells were treated according to the scheme shown in Figure S2a.

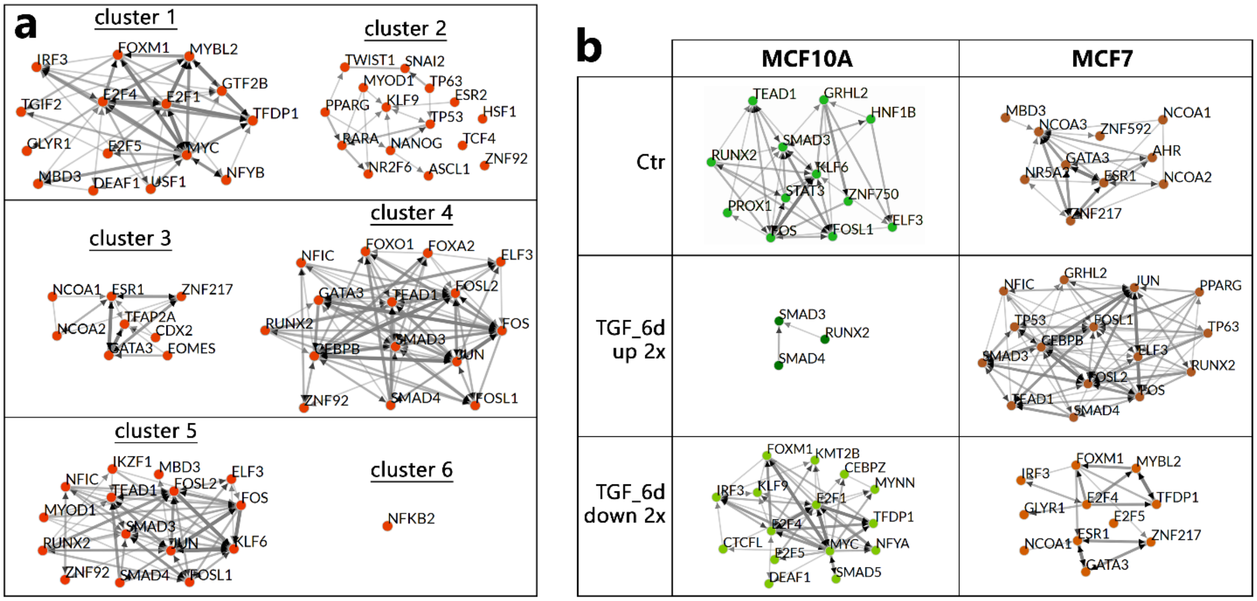

Figure S4. Networks of upstream regulators (predicted using the ChEA3_ReMap) of genes identified by RNA-seq analyses in TGFB1-treated MCF10A and MCF7 cells. (**a**) Transcription factors associated with gene sets from clusters 1-6 shown in Figure 1c and Supplementary Data 2. (**b**) Transcription factors associated with genes differentiating untreated (Ctr) MCF10A and MCF7 cells (10-fold difference), stimulated (up 2x), or repressed (down 2x) on the sixth day of TGFB1 stimulation (TGF_6d). FET p-value < 0.05; the top 15 factors are shown.

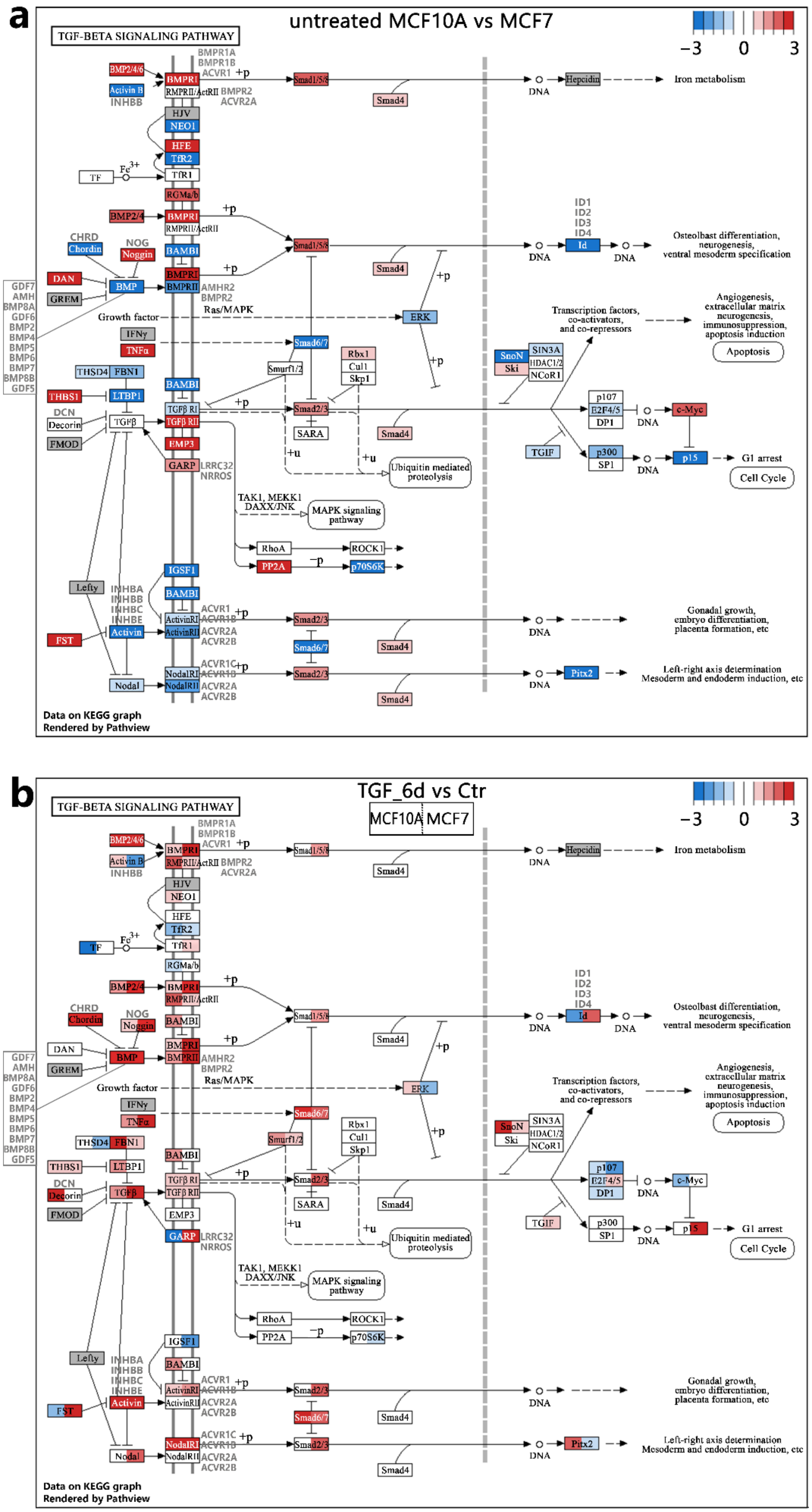
a
untreated MCF10A vs MCF7
TGF-BETA SIGNALING PATHWAY
−3 0 3
BMP2/4/6
Activin B
INHBB
BMPRI
RMPRII/ActRII
BMPR1A
BMPR1B
ACVR1
BMPR2
ACVR2A
+p
Smad1/5/8
Smad4
DNA
Hepcidin
Iron metabolism
HJV
NEO1
HFE
TfR2
TfR1
TF
Fe^{3+}
RGMa/b
BMP2/4
CHRD
Chordin
NOG
Noggin
BAMBI
DAN
GREM
BMP
BMPRII
AMHR2
BMPR2
GDF7
AMH
BMP8A
GDF6
BMP2
BMP4
BMP5
BMP6
BMP7
BMP8B
GDF5
ID1
ID2
ID3
ID4
Id
Osteoblast differentiation, neurogenesis, ventral mesoderm specification
Growth factor
Ras/MAPK
ERK
IFNγ
TNFα
Smad6/7
Transcription factors, co-activators, and co-repressors
Angiogenesis, extracellular matrix neurogenesis, immunosuppression, apoptosis induction
Apoptosis
THSD4
FBN1
Smurf1/2
Rbx1
Cul1
Skp1
SnoN
Ski
SIN3A
HDAC1/2
NCoR1
THBS1
LTBP1
DCN
Decorin
TGFβ
TGFβ RI
TGFβ RII
Smad2/3
SARA
p107
E2F4/5
DP1
c-Myc
FMOD
EMP3
GARP
LRRC32
NRROS
+u
Ubiquitin mediated proteolysis
TGIF
p300
SP1
p15
G1 arrest
Cell Cycle
TAK1, MEKK1
DAXX/JNK
MAPK signaling pathway
RhoA
ROCK1
PP2A
−p
p70S6K
IGSF1
Lefty
INHBA
INHBB
INHBC
INHBE
ActivinRI
ActivinRII
ACVR1
ACVR1B
ACVR2A
ACVR2B
FST
Activin
Gonadal growth, embryo differentiation, placenta formation, etc
NodalRI
NodalRII
ACVR1C
Nodal
Pitx2
Left-right axis determination
Mesoderm and endoderm induction, etc
Data on KEGG graph
Rendered by Pathview
b
TGF_6d vs Ctr
MCF10A MCF7
TGF-BETA SIGNALING PATHWAY
−3 0 3
Data on KEGG graph
Rendered by Pathview

Figure S5. Comparison of the TGF-beta signaling pathway in MCF10A and MCF7 cells. (**a**) Comparison of the transcript levels in untreated cells. Bar scale legend is log2 fold change in MCF10A vs MCF7 (red means higher expression in MCF10A, blue – in MCF7). (**b**) Comparison of log2-fold changes on the sixth day of TGFB1 stimulation in MCF10A (left part of each rectangle) and MCF7 (right part of each rectangle) cells. Gene expression data from the RNA-seq experiment were mapped to the TGF-beta signaling pathway in the KEGG database (ID: hsa04350; https://www.kegg.jp/pathway/hsa04350). Colors indicate gene up-regulation (red) and down-regulation (blue); grey indicates the missing data. The color scale was truncated to values within the range of −3 to 3. The regular map notation is available at https://www.kegg.jp/kegg/document/help_pathway.html.

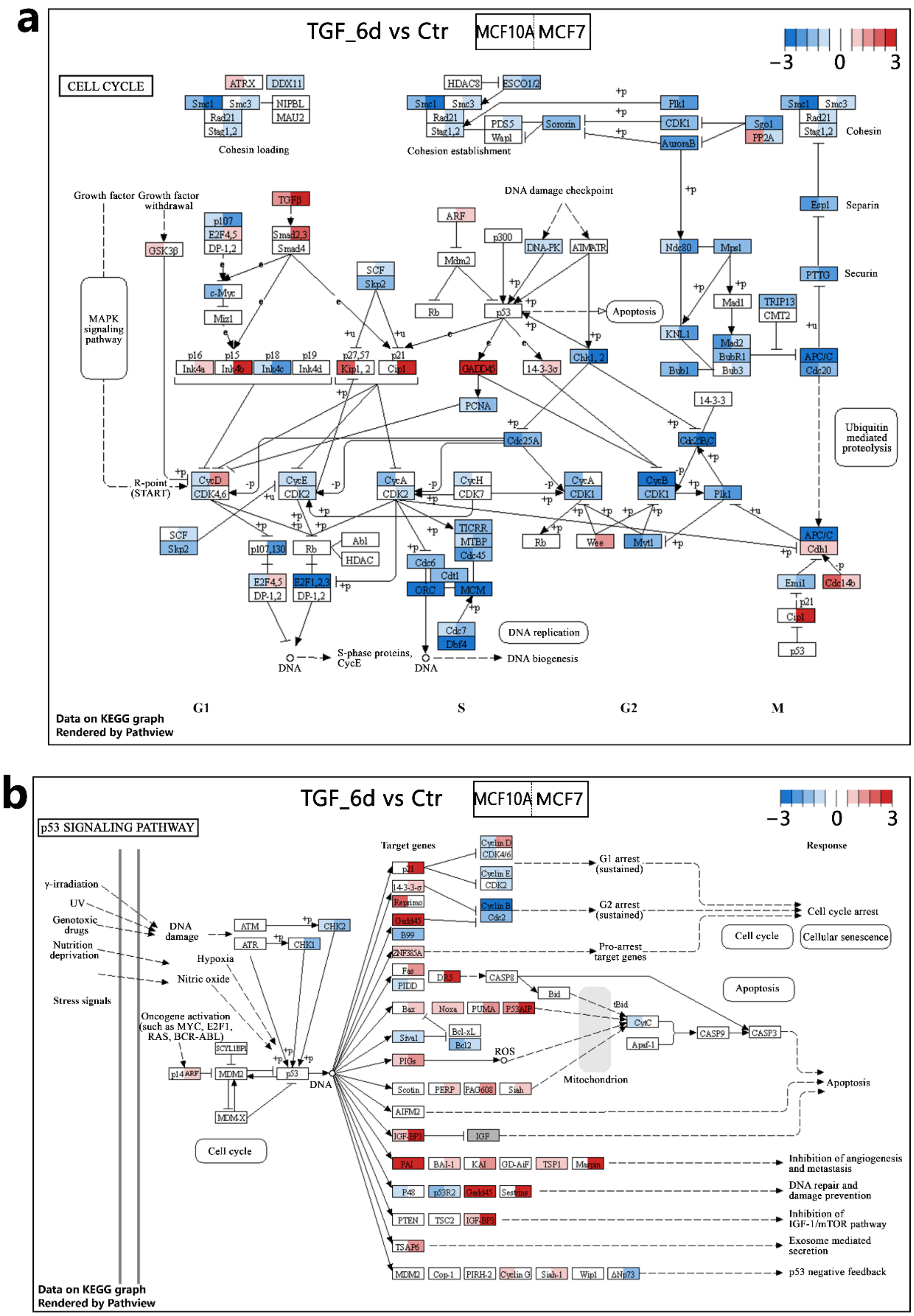

Figure S6. Changes in expression of genes related to the cell cycle network (**a**) and the p53 signaling pathway (**b**) on the sixth day of TGFB1 stimulation in MCF10A (left part of each rectangle) and MCF7 (right part of each rectangle) cells. Log2-fold changes from the RNA-seq experiment were mapped to the KEGG pathways: Cell cycle (ID: hsa04110; https://www.kegg.jp/pathway/hsa04110) and p53 signaling pathway (ID: hsa04115;

https://www.kegg.jp/pathway/hsa04115). Colors indicate gene upregulation (red) and downregulation (blue); grey indicates the missing data. The color scale was truncated to values within the range of −3 to 3. The regular map notation is available at https://www.kegg.jp/kegg/document/help_pathway.html.

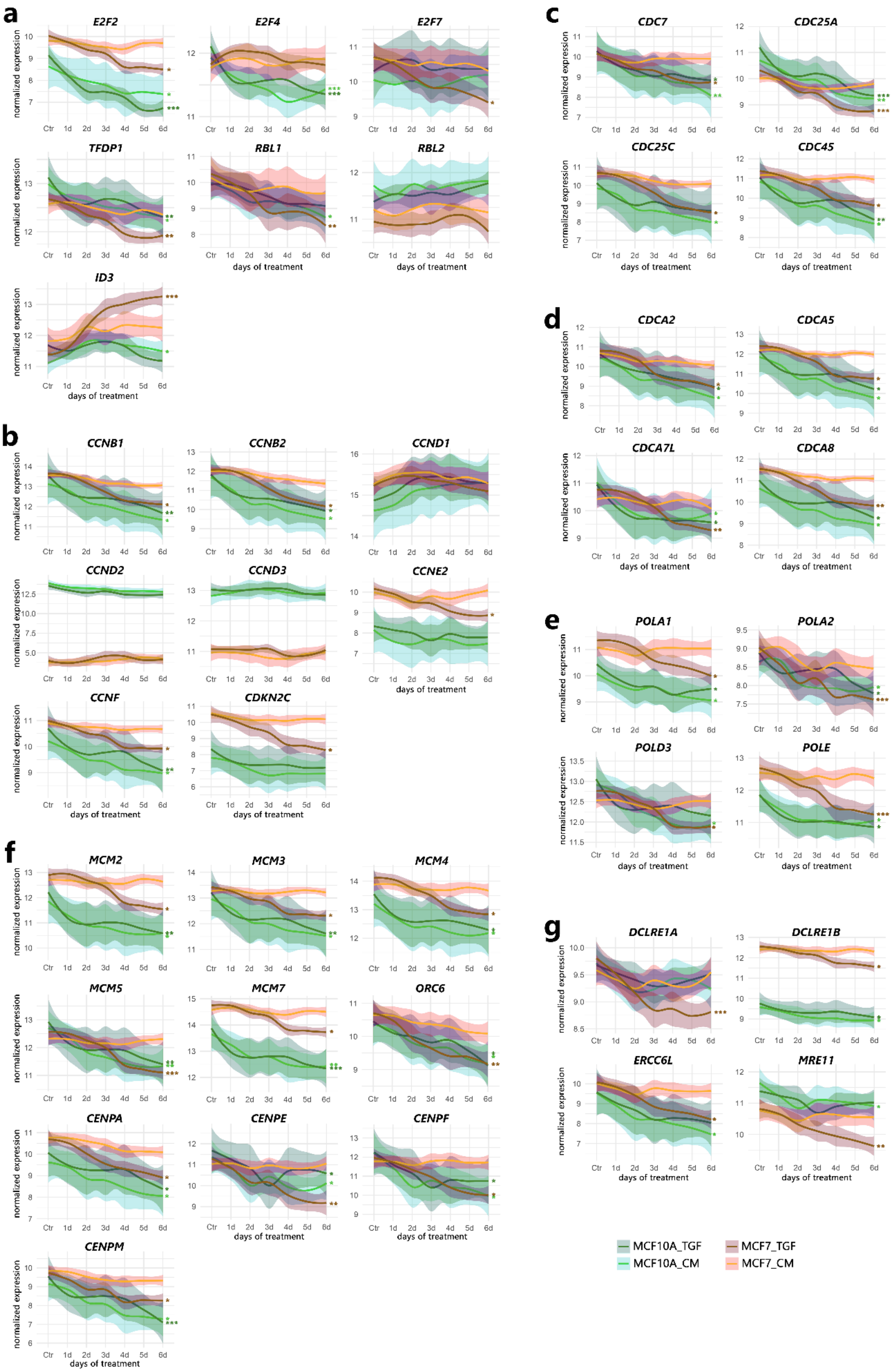
a
E2F2
E2F4
E2F7
TFDP1
RBL1
RBL2
ID3
b
CCNB1
CCNB2
CCND1
CCND2
CCND3
CCNE2
CCNF
CDKN2C
c
CDC7
CDC25A
CDC25C
CDC45
d
CDCA2
CDCA5
CDCA7L
CDCA8
e
POLA1
POLA2
POLD3
POLE
f
MCM2
MCM3
MCM4
MCM5
MCM7
ORC6
CENPA
CENPE
CENPF
CENPM
g
DCLRE1A
DCLRE1B
ERCC6L
MRE11
normalized expression
days of treatment
Ctr 1d 2d 3d 4d 5d 6d
MCF10A_TGF
MCF10A_CM
MCF7_TGF
MCF7_CM

Figure S7. Time trends in the expression of genes related to cell cycle progression in MCF10A and MCF7 cells. Examples of: (**a**) transcriptional regulators, (**b**) cyclins and inhibitor of cyclin-dependent kinase *CDKN2C*, (**c**) cell division cycle proteins, (**d**) cell division cycle associated proteins, (**e**) DNA polymerases, (**f**) proteins involved in chromosomal replication, and (**g**) proteins involved in DNA repair. Cells were treated according to the scheme shown in Figure S2a. TGF, direct TGFB1 treatment; CM, conditioned medium treatment. *** padj < 0.0001, ** padj < 0.001, * padj < 0.05 (significance of differences was marked for the entire run if the adjusted p value vs Ctr reached a given value at least at one experimental point). Supplementary to Figure 4.

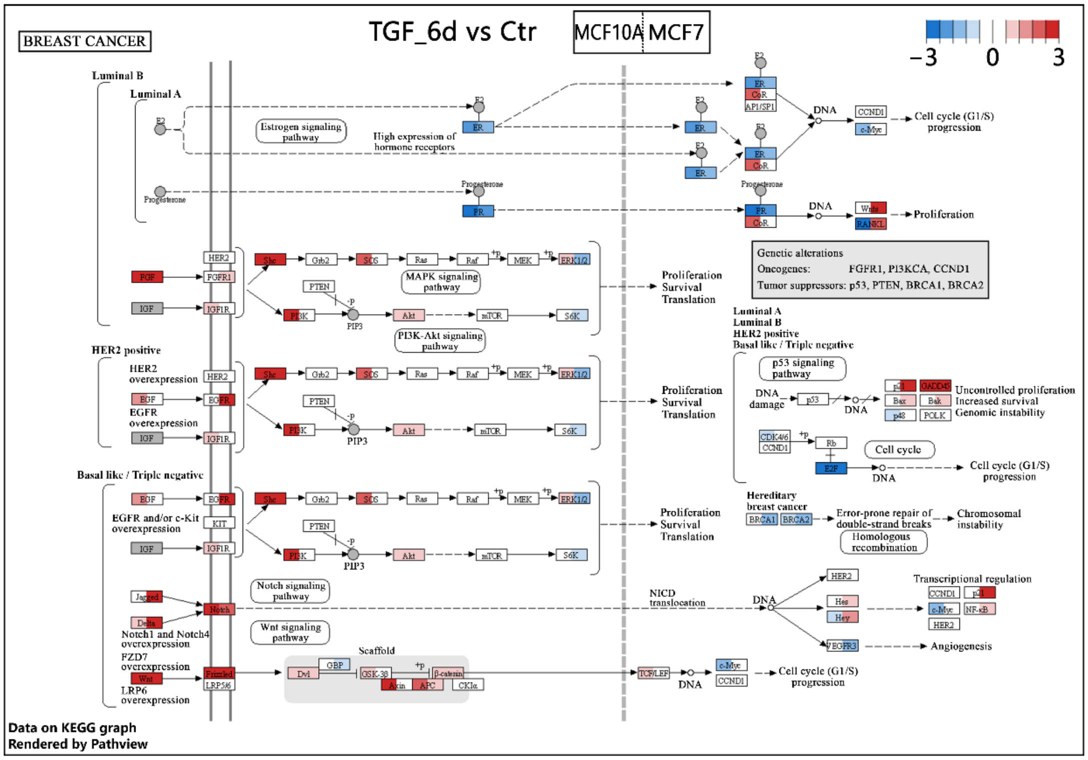

Figure S8. Changes in expression of genes related to the breast cancer network on the sixth day of TGFB1 stimulation in MCF10A (left part of each rectangle) and MCF7 (right part of each rectangle) cells. Log2-fold changes from the RNA-seq experiment were mapped to the Breast Cancer network (ID: hsa05224; https://www.kegg.jp/pathway/hsa05224) in the KEGG database. Colors indicate gene upregulation (red) and downregulation (blue); grey indicates the missing data. The color scale was truncated to values within the range of −3 to 3. The regular map notation is available at https://www.kegg.jp/kegg/document/help_pathway.html.

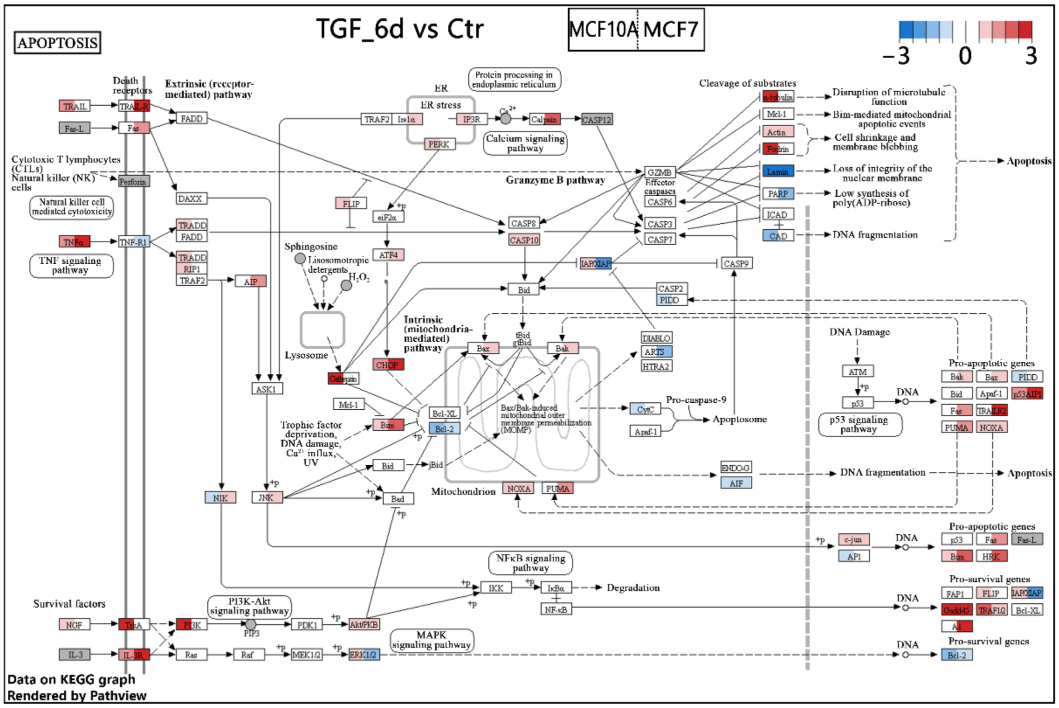

Figure S9. Changes in expression of genes related to the apoptosis network on the sixth day of TGFB1 stimulation in MCF10A (left part of each rectangle) and MCF7 (right part of each rectangle) cells. Log2-fold changes from the RNA-seq experiment were mapped to the Apoptosis network (ID: hsa04210; https://www.kegg.jp/pathway/hsa04210) in the KEGG database. Colors indicate gene upregulation (red) and downregulation (blue); grey indicates the missing data. The color scale was truncated to values within the range of −3 to 3. The regular map notation is available at https://www.kegg.jp/kegg/document/help_pathway.html.

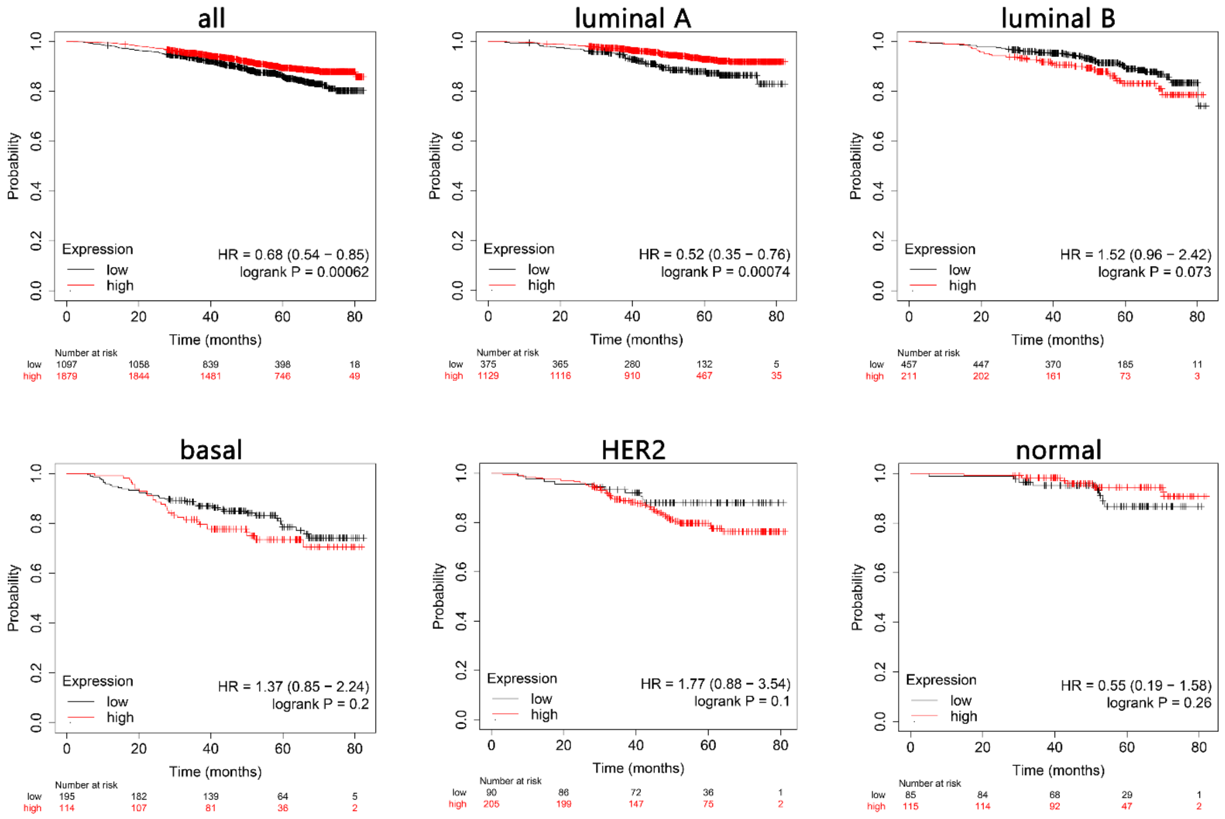

Figure S10. Effect of *TGFB1* transcript levels on overall survival in breast cancer patients analyzed using the Kaplan Meier Plotter available at https://kmplot.com/analysis/index.php?p=service&cancer=breast_rnaseq_gse96058 (Győrffy 2021) (accessed March 2024). Patients (n=2976) were split by "Auto select best cutoff". All patients were analyzed (all) or analysis was restricted to PAM50 subtypes: luminal A (n=1,504), luminal B (n=668), basal (n=309), HER2 (n=295), and normal (n=200).
