# Supplementary material for: Transcriptional responses to direct and indirect TGFB1 stimulation in cancerous and noncancerous mammary epithelial cells
Source: Cell Commun Signal. 2024 Oct 28;22:522. doi: 10.1186/s12964-024-01821-5 (PMC11514872; doi:10.1186/s12964-024-01821-5)

Transcriptional responses to direct and indirect TGFB1 stimulation in cancerous and noncancerous mammary epithelial cells.

Original blots

Fig 5b ACTB:

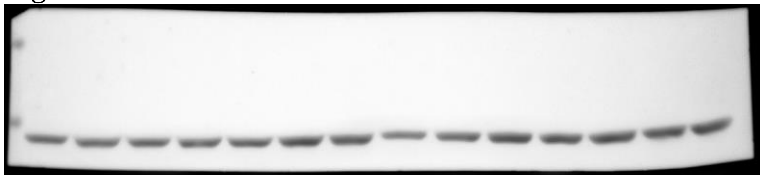

Fig 5b ACTB_ESR1:

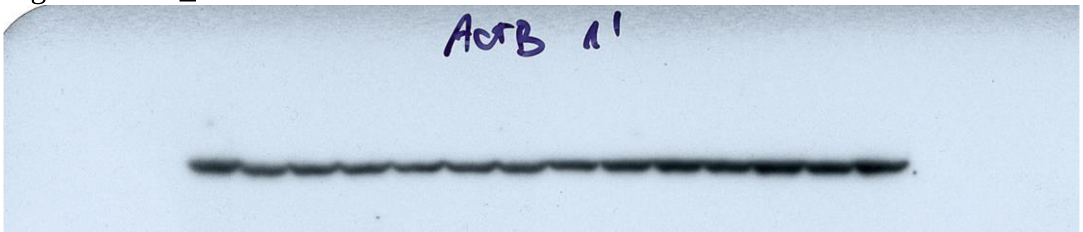

Fig 5b ACTB_VIM_CASP7:

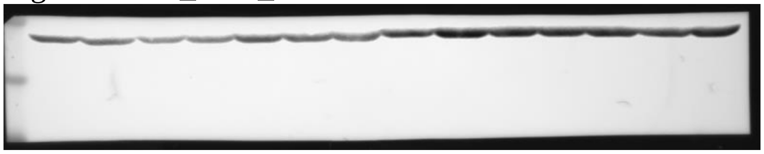

Fig 5b CASP7 cleaved:

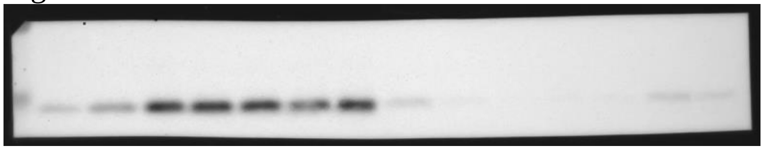

Fig 5b CASP9 cleaved:

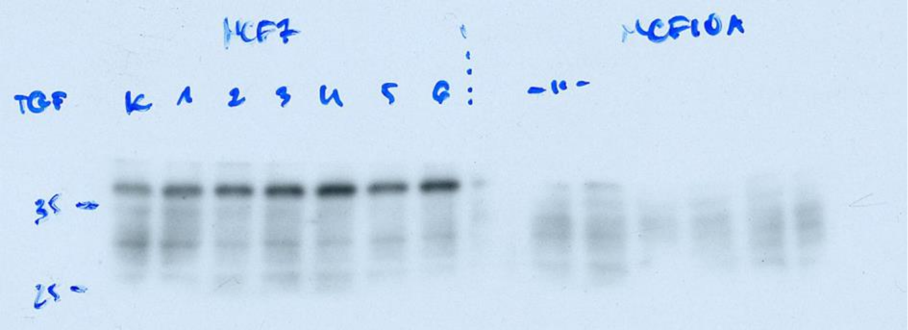

Fig 5b CDH1:

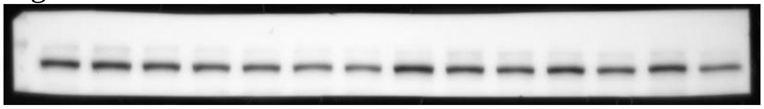

Fig 5b ESR1:

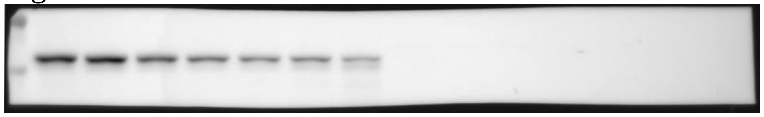

Fig 5b PARP cleaved:

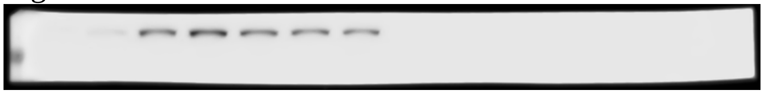

Fig 5b P-SMAD3:

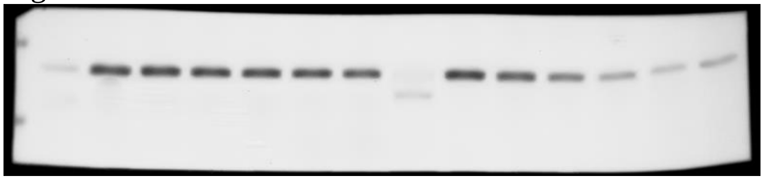

Fig 5b VIM:

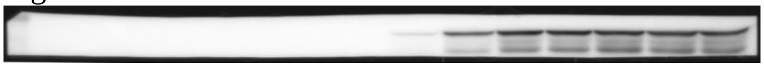

Fig S1a CDH1:

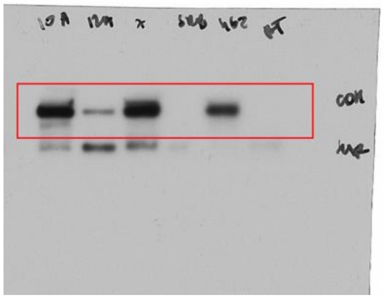

Fig S1a HSPA8:

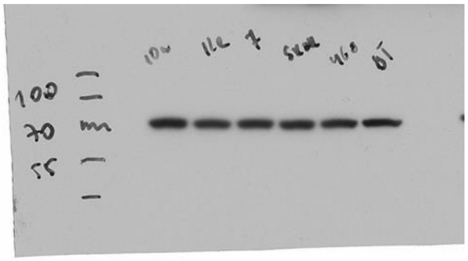

Fig S1b ACTB_CM:

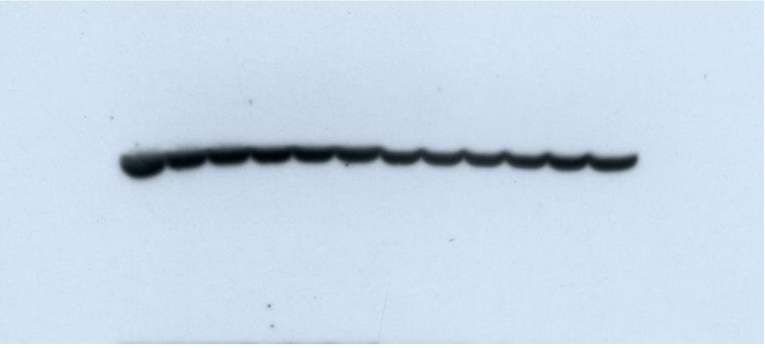

Fig S1b ACTB_TGFB1:

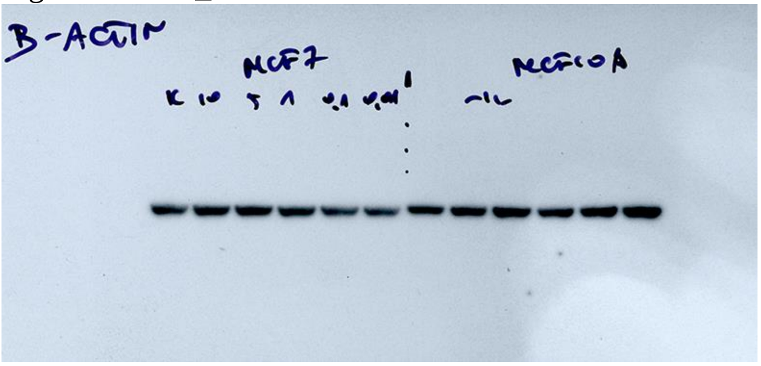

Fig S1b p-SMAD3_CM:

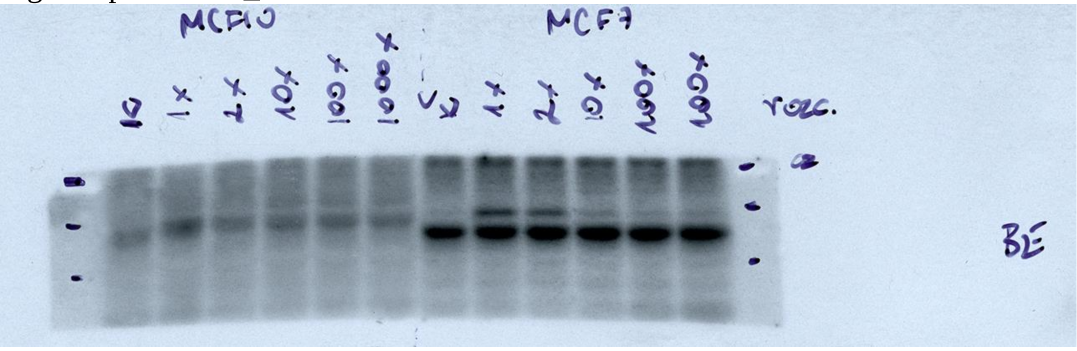

Fig S1b p-SMAD3_TGFB1:

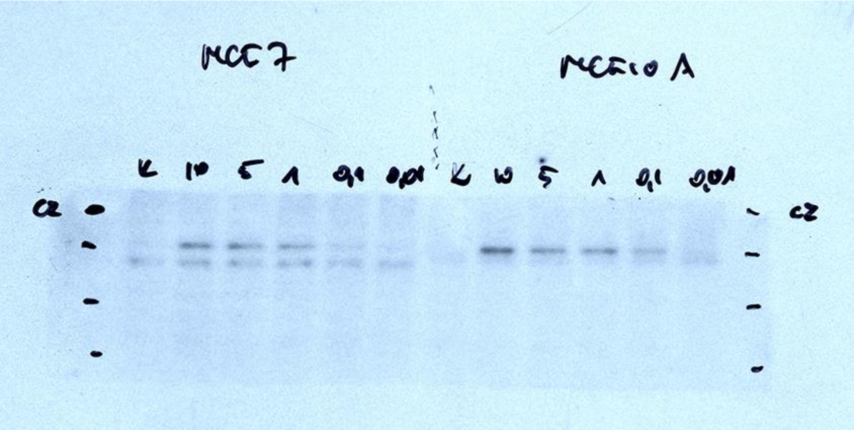

Fig S1c ACTB:

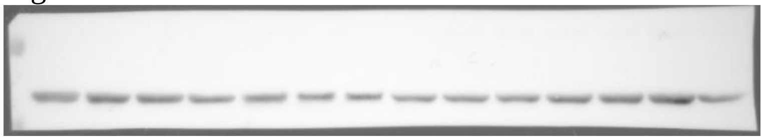

Fig S1c BRD4:

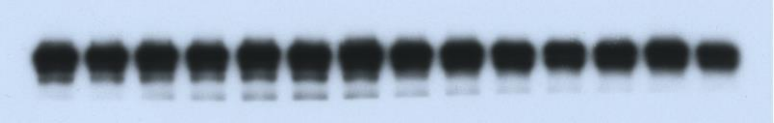

Fig S1c cleaved PARP1:

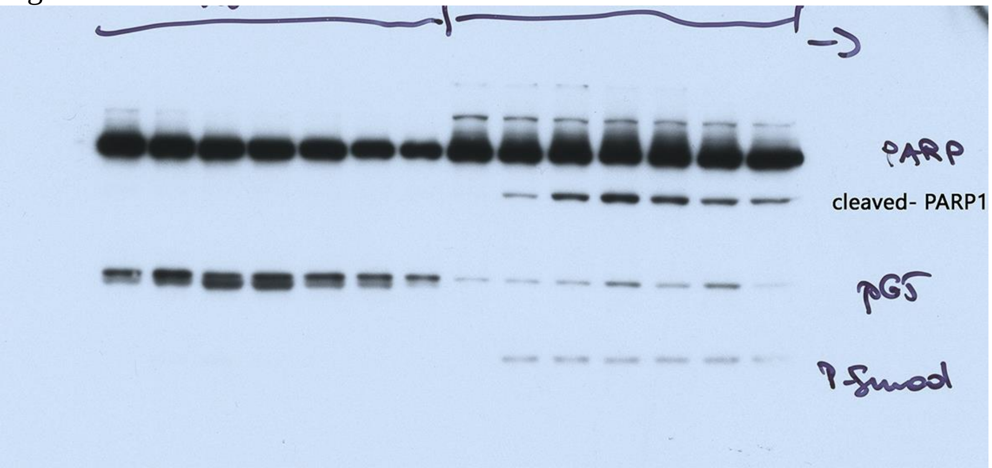

Fig S1c PARP1:

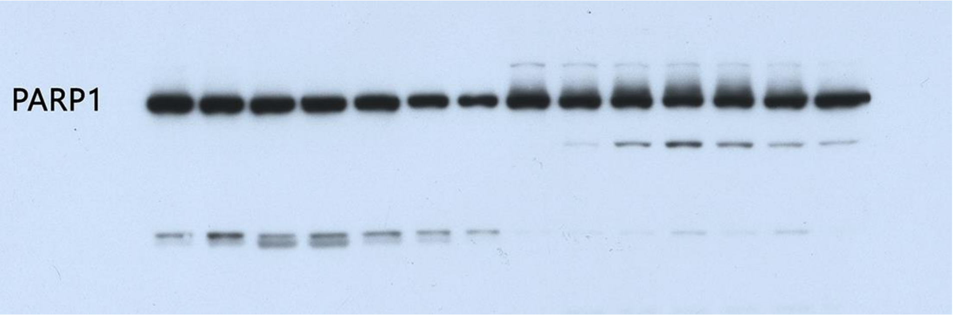

Fig S1c SNAI1 and p-SMAD3:

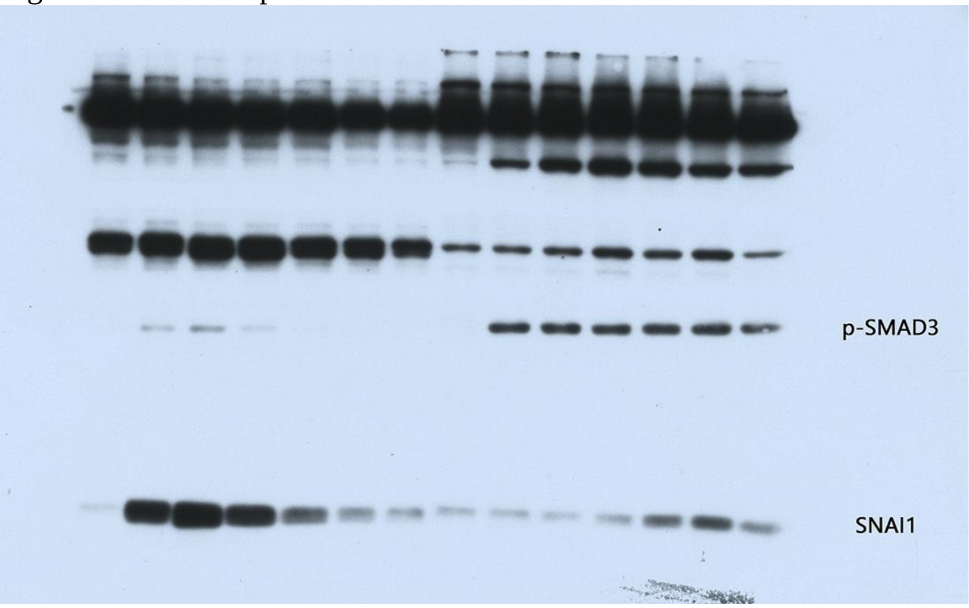

Fig S1d MCF7_ACTB:

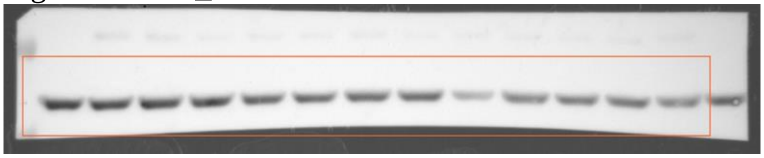

Fig S1d MCF7_BRD4, PARP1 and cleaved PARP1:

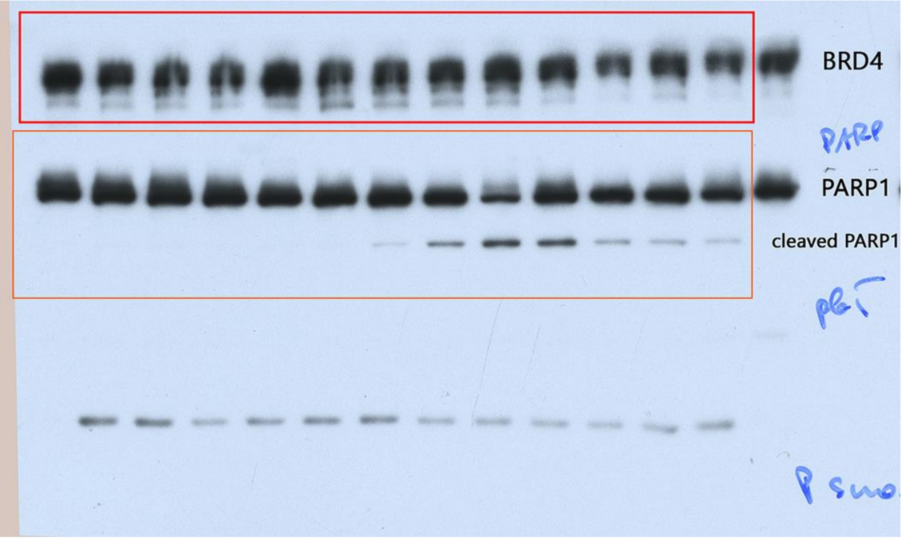

Fig S1d MCF7_p-SMAD3:

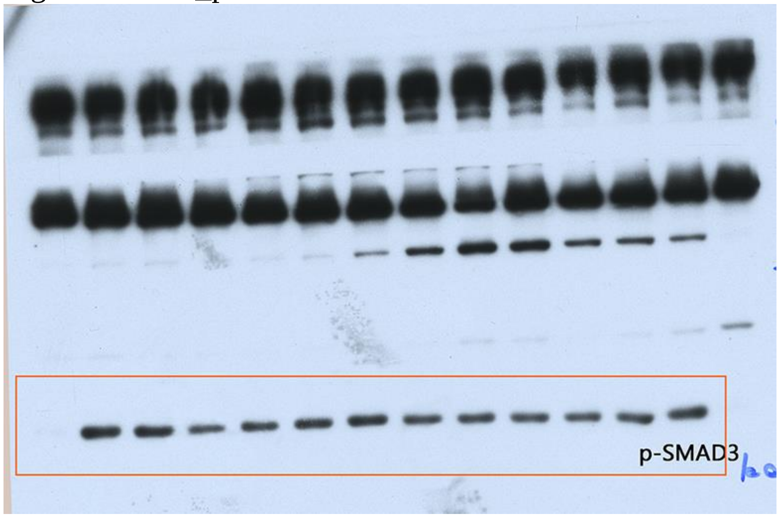

Fig S1d MCF10A_ACTB:

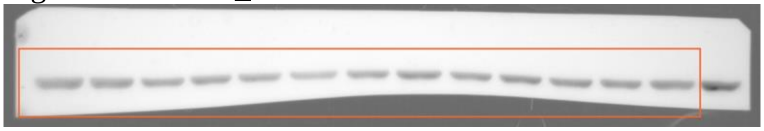

Fig S1d MCF10A_p-SMAD3:

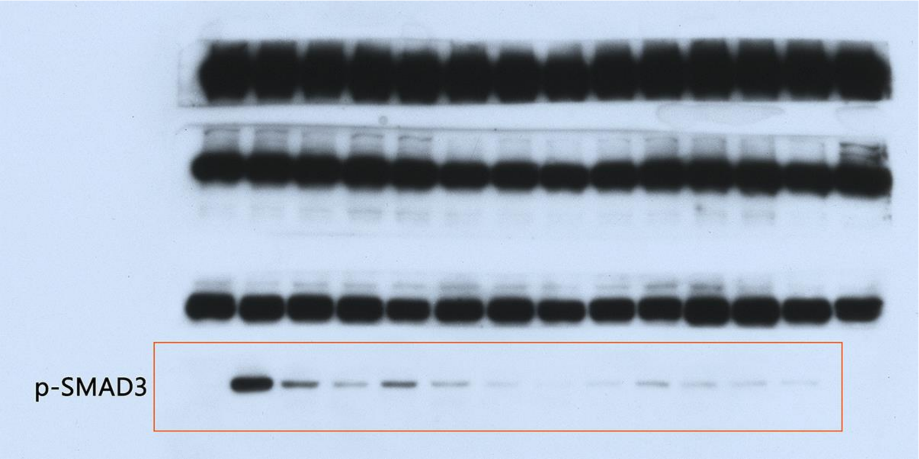

Fig S1d MCF10A_SNAI1:

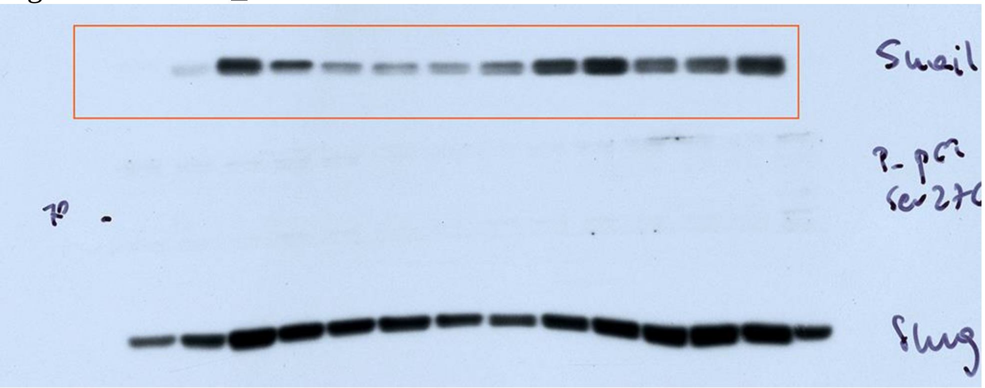

Fig S1d MCF10A_SNAI2:

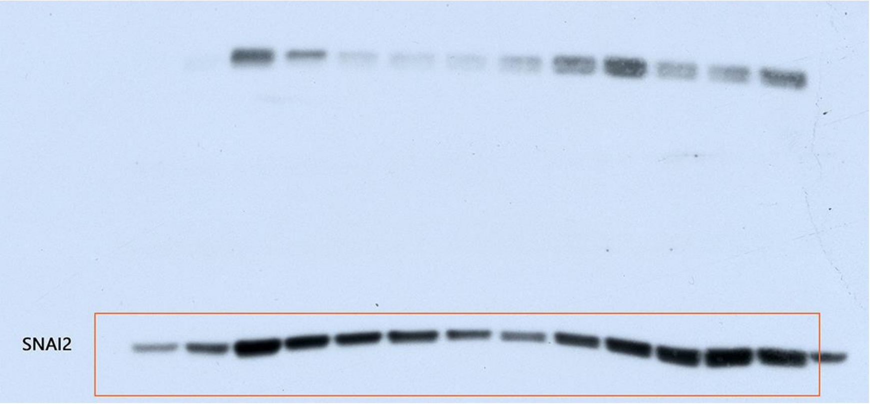

Fig S1d_MCF7_SNAI1 and SNAI2:

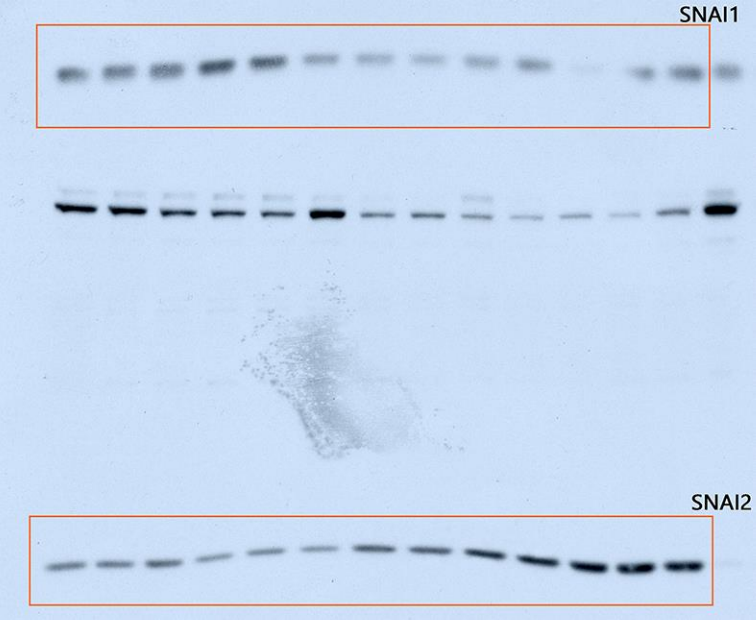

Fig S1d_MCF10A_BRD4, PARP1 and cleaved PARP1:

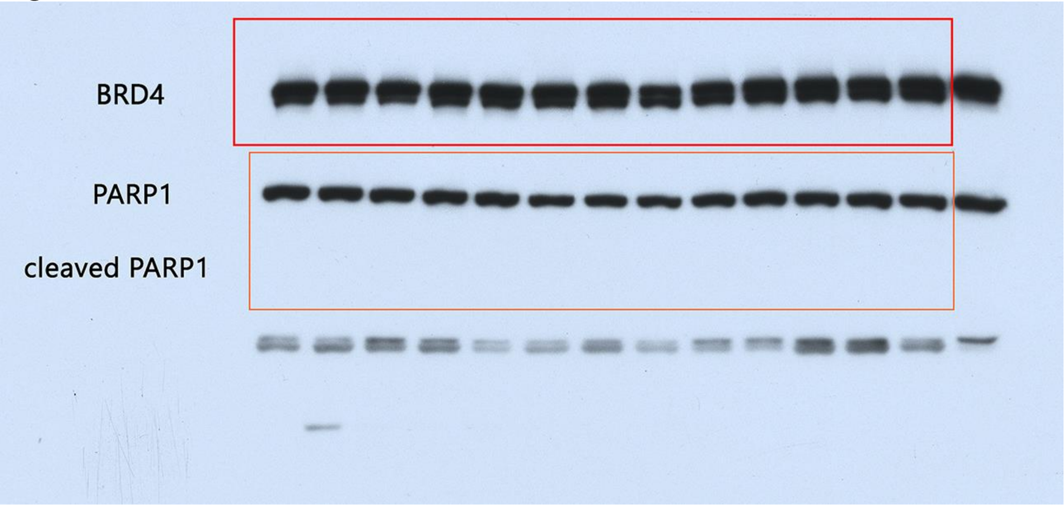

Supplement: Supplementary file 5 — Supplementary Material 5: Original blots [file 12964_2024_1821_MOESM5_ESM.pdf]
